# Approximation of a Microbiome Composition Shift by a Change in a Single Balance Between Two Groups of Taxa

**DOI:** 10.1128/msystems.00155-22

**Published:** 2022-05-09

**Authors:** Vera E. Odintsova, Natalia S. Klimenko, Alexander V. Tyakht

**Affiliations:** a Atlas Biomed Group–Knomx LLC, Moscow, Russia; b Center for Precision Genome Editing and Genetic Technologies for Biomedicine, Institute of Gene Biology, Russian Academy of Sciences, Moscow, Russia; University of California San Diego

**Keywords:** compositional analysis, balances, microbial signatures, microbiome, metagenomics, principal balance analysis, regression analysis

## Abstract

Linking microbiome composition obtained from metagenomic or 16S rRNA sequencing to various factors poses a real challenge. The compositional approach to such data is well described: a so-called isometric log-ratio (ILR) transform provides correct treatment of relative abundances. Most existing compositional methods differ in the particular choice of the transform. Although this choice does not influence the prediction of a model, it determines the subset of balances between groups of microbial taxa subsequently used for interpreting the composition shifts. We propose a method to interpret these shifts independently of the initial choice of ILR coordinates by the nearest single-balance shift. We describe here application of the method to regression, classification, and principal balance analysis of compositional data. Analytical treatment and cross-validation show that the approach provides the least-squares estimate of a single-balance shift associated with a factor with possible adjustment for covariates. As for classification and principal balance analysis, the nearest balance method provides results comparable to other compositional tools. Its advantages are the absence of assumptions about the number of taxa included in the balance and its low computational cost. The method is implemented in the R package NearestBalance.

**IMPORTANCE** The method proposed here extends the range of compositional methods providing interpretation of classical statistical tools applied to data converted to the ILR coordinates. It provides a strictly optimal solution in several special cases. The approach is universally applicable to compositional data of any nature, including microbiome data sets.

## INTRODUCTION

The development of high-throughput DNA sequencing methods has provided vast opportunities to explore complex microbial communities inhabiting various ecological niches such as soil, ocean, and host-associated locations. Hence, it has become particularly important to standardize microbiome data analysis at each step. To date, there is no golden standard of statistical approach to these data. Researchers have to choose a single method from a wide assortment (from the nonparametric Mann-Whitney test to complex probabilistic models) in order to overcome the limitations arising from the specific nature of microbiome composition data, such as compositionality and sparsity.

Generally, each statistical method for microbiome data implements one of two approaches: component-wise or compositional. Component-wise methods treat each taxon individually and differ by their underlying probabilistic models. On the other hand, compositional methods treat each species as a part of the whole community and differ by the specific ways to identify patterns in the composition. Several reviews (1, 2) emphasized that only the compositional approach is appropriate for the analysis of proportions, while the component-wise approach leads to biases and unreliable conclusions. However, component-wise methods are still widespread since they are more intuitive and simpler. They yield a list of taxa associated with a factor. In contrast, most compositional methods yield a list of balances: the proportions between groups of taxa calculated in a specific way. As such, this approach requires more efforts to achieve meaningful interpretations for expert biologists and a wider audience.

Here, we propose a method to interpret the results of compositional analysis. This approach aims to approximate the list of balances by a single balance. To make the idea clearer, let us review some details of component-wise approach and current compositional methods (a more rigorous and complete review can be found in reference 3).

In most cases, 16S rRNA sequencing does not give information about the absolute amount of taxa in a sample but only the number of reads mapped on the genome of each taxa from a database (counts). All information contained in the data are related to the proportions between taxa. This sort of information is similar to a recipe for a drink: it may say to mix 50 mL of espresso, 40 mL of milk, and 10 mL of syrup, but making a double portion (100 mL of espresso, 80 mL of milk, and 10 mL of syrup) actually does not change the taste, since it is only the proportions between components that matter. Such data, i.e., providing a vector with positive numbers for which only proportions between components are informative and where neither the order nor the total sum are important, are called a composition. Data obtained from sequencing are compositional.

**FIG 1:**
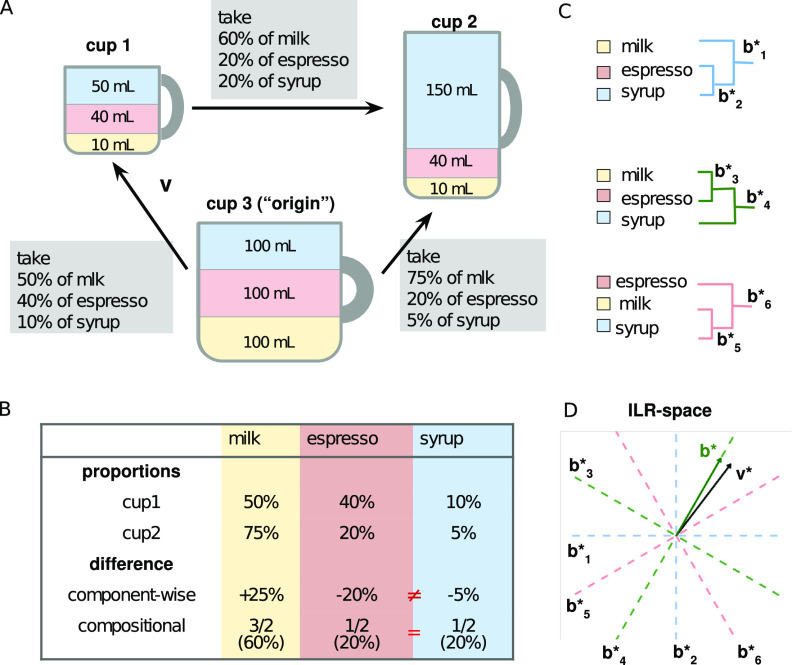
Compositional approach to the analysis of difference between two compositions: an example of two coffee recipes. (A) Definition of perturbation as a fold change of components’ proportions in samples. (B) Comparison of compositional and component-wise definitions of the difference in proportions. (C) Binary trees that may be used for the construction of an ILR system of coordinates. The difference between the drinks may be described by a shift in balance b*_1_, while b*_2_ is constant across the compositions. (D) Visualization of the suggested method: search for a balance b that is the closest to some vector of interest v in an ILR system of coordinates [v* = ILR(v), b* = ILR(b)].

The most obvious disadvantage of the component-wise approach to such data are missing the interdependence of components’ relative proportions. Figure 1 illustrates the intuition of this disadvantage by comparing two coffee drinks. Consider two cups of coffee that both include syrup, espresso, and milk. The only difference between them is that the second cup contains a triple portion of milk: it is the first cup with the addition of two milk portions (Fig. 1A). If one were to taste a spoonful of each drink, the observed dissimilarities would refer to each component: the second drink would be less sweet, less strong, and more milky. While that assessment would be accurate, a more informative answer would be that the proportions between coffee and syrup in the spoons are the same, and it is only the proportion of milk to the other ingredients that differs. Now, let us imagine a study comparing the gut microbiome compositions of healthy subjects and those affected by a certain disease. If the sample size is large enough, some differences in the proportions of all taxa may be observed even if only a single pathogenic taxon overgrows in the patients. As with the coffee example, those differences would be true, but a more important conclusion would be that the commensal microbiome remained unperturbed and that only the proportion of a specific pathogen to the other species changed. However, an insufficient sample size may lead to additional bias, whereby the list of the affected taxa would be shorter and would depend on their initial proportions, as the effect size of observed changes varies between individual species. Nonetheless, it would not imply that some commensal taxa increased in presence, whereas others did not increase at all or did so to a lower extent. Figure 1B illustrates this bias on the coffee example: although the absolute change between the cups is equally absent for both syrup and espresso, the component-wise approach detects differences between changes in the proportions of these two components; the observed change in espresso proportion is almost as high as for the milk, even though milk was the only altered component.

The compositional approach starts from changing the perspective on what a taxon’s differential presence between two samples means: it is the ratio of relative abundances in the two samples, rather than the difference. Returning to the coffee example, the fold changes of coffee and syrup are the same and differ from the fold change in milk proportion (Fig. 1B); such a description of dissimilarities is more informative. This kind of difference, thought of as a shift that should be applied to the first sample to obtain the second one, is called a “perturbation.”

Interestingly, a perturbation itself may be treated as a composition: only the proportions of its components matter, and normalization to, for instance, 100%, preserves the result. The reverse is also true: composition may be thought of as a perturbation of an “origin,” a composition with equal proportions of all components (Fig. 1A). This initial composition, when thought of as a perturbation, represents a zero shift (leaving one-third of all milk, espresso, and syrup does not change the taste of coffee), making this definition similar to the one of a zero vector in a vector space.

Thus, we have a vector space of compositions with a natural definition of the origin and the summation (adding a perturbation). This space may be accomplished with specific definitions of the inner product and Aitchison distance that estimates the differences in proportions between two samples. Together, they represent a Euclidean space (3). Though the Euclidean geometry is quite intuitive, in our specific case operations with vectors are inconvenient (for example, component-wise division is not a natural way of calculating the difference); it obstructs usage of such common methods as linear regression or linear discriminant analysis.

A solution to the problem has been presented in reference 4. The authors of that study proposed the so-called isometric log-ratio (ILR) transform; this preserves the results of major operations with vectors (e.g., inner product and compositional difference) and converts data to a new space, which allows for further calculations to occur in a natural way. Following the “principle of working in coordinates” (5), the data should be transformed to ILR coordinates, all the analysis should be performed there, and then the inverse conversion should be applied to the results for interpretation.

Such ILR transform may be defined in numerous ways: the rotation of the basis around the origin preserves all required properties. The choice of particular ILR coordinates does not influence the results of the analysis when they are transformed back to the initial space of proportions. This approach is convenient for interpretation of vectors that are treated as compositions rather than perturbations. For example, microbiome composition predicted by linear regression in the ILR coordinates can be easily transformed to a vector of taxa proportions. The vector of differences between two samples is harder to interpret in the initial terms. Its meaning is less intuitive: it describes which proportion of each taxon from the first sample should be taken to obtain the microbiome with the proportions observed in the second sample (Fig. 1A).

In reference 6, the authors suggested a method to define an interpretable system of coordinates. It is based on the idea of using simple perturbations as coordinate vectors. Imagine adding the second, third, and fourth portions of milk to a cup of coffee and leaving the syrup/espresso ratio unperturbed. This may be viewed as perturbations of the initial state in the same direction with increasing effect size (only one component of perturbation will change). Thus, compositions of the coffee will shift along a straight line in the ILR space. The idea may be generalized to a more complex case of perturbing proportions between two groups of taxa and leaving unperturbed all other independent proportions. This relation between two groups is called a “balance” and should be calculated in a specific way: it is a proportion between the abundances of the “mean” representative of each group of taxa. The “mean” is understood as a geometric mean, the proportion is treated in logarithmic scale to make it symmetric relative to the choice of groups in the numerator and in the denominator (see details in Materials and Methods). The basis of the ILR coordinate system is constructed of simple unit perturbations called balancing elements. The choice of orthogonal balancing elements is based on a binary tree with components as leaves; each node of the tree is used to define a balance between two groups of taxa represented by the leaves of its two branches (Fig. 1C). Now, any shift of microbiome can be described as several simple perturbations of known effect size.

The binary tree may be constructed in many ways; the specific choice limits the set of simple perturbations used for interpretation of changes in microbiome. Construction of the tree may be based on the phylogenetic affinity of taxa (7), approximate results of principal coordinates analysis (8, 9), or a difference in phenotype between samples (9, 10).

Almost all compositional methods assume that the choice of the coordinates is made before the statistical analysis. In a more complicated algorithm, the choice of just one balance and the analysis are iterated many times to find the ultimate balance that provides the best accuracy (*selbal* algorithm [11]).

Here, we propose to reverse the order: first, perform the analysis in any arbitrary ILR system of coordinates and only then look for interpretation. We present an algorithm (here called “the nearest balance method”) that finds the nearest interpretable direction to the vector of interest. The output format of the algorithm is very close to the results provided by component-wise analysis: a list of taxa positively or negatively associated with a factor.

Figure 1D illustrates how the interpretation is obtained for the coffee example. The first cup differs from the “origin” by a complex shift “v” which cannot be described by a change in a single balance (Fig. 1A). An ILR transform converts proportions to a plane (Fig. 1D); any pair of balances constructed by a binary tree (Fig. 1C) corresponds to two orthogonal directions in this plane and may be used as a coordinate system. A simple procedure described in Materials and Methods allows us to find the balance defining the direction closest to the vector of differences. Here, such a balance is the proportion of milk and coffee to syrup.

**FIG 2:**
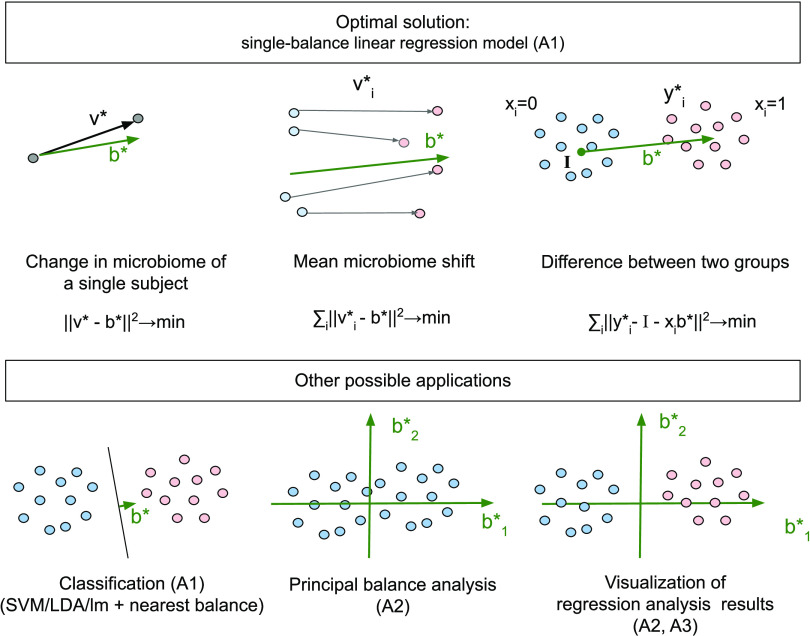
Examples of applications of the nearest balance approach. Here, ‖.‖ denotes Euclidean norm in the ILR space (which equals the Aitchison norm for the proportions). A1, A2, and A3 denote the applied algorithms from the Materials and Methods section.

The proposed algorithm gives an exact solution. In addition, we describe here two similar algorithms: (i) the construction of an ILR system of coordinates based on the nearest balance principle and (ii) the construction of two orthogonal balances for interpreting two ILR vectors. These three algorithms may be combined with any statistical method that provides ILR vectors as a result. Examples of applications (Fig. 2) include interpreting and visualization of various statistical methods’ results, such as classification, linear regression, and principal component analysis.

Importantly, the method provides the nearest balance to a microbiome shift vector but does not guarantee that this balance is the best in terms of the initial optimization problem, such as minimization of the loss.

However, we show below that the approach provides a least-squares solution for several simple but useful cases. First of all, it is obviously a least-squares approximation of a single subject microbiome shift. To our knowledge, this is the first algorithm to facilitate the interpretation of individual changes. Next, nearest balance provides a least-squares solution for a linear regression model with one of the predictors being associated with a single-balance shift (“single-balance regression”). The method provides accuracy comparable to existing compositional methods for the classification problem and “principal balance analysis” (the search for a sequence of orthonormal balances successively maximizing the explained variance in a data set [8]).

## RESULTS

Here, we propose three algorithms, which are described in detail in Materials and Methods.

The first algorithm (A1) provides the nearest balance “b” to a given microbiome composition shift “v.” The second algorithm (A2) is designed for joint interpretation of two ILR-vectors (v_1_, v_2_) (such as two first PCA components) by two orthogonal balances (b_1_ is the nearest to v_1_; b_2_ is the nearest balance to v_2_ among all balances orthogonal to b_1_). The third algorithm (A3) provides an ILR system of coordinates to decompose a microbiome shift v into a superposition of orthogonal balances (b_1_, b_2_, …, b*_D_*_-1_) sequentially approximating the shift.

The presentation here is organized as follows. In Results we explore several applications: interpretation of individual changes in microbiome composition (A1), regression analysis (A1 for analysis, A3 for validation), classification (A1 for analysis, A2 for visualization, A3 for validation), and principal balance analysis (A2). The Materials and Methods section describes algorithms in detail and contains the proof of optimality for certain applications. The Discussion section summarizes the advantages and disadvantages of the approach and describes directions for future improvements.

### Interpretation of single subjects’ microbiome composition changes.

**FIG 3:**
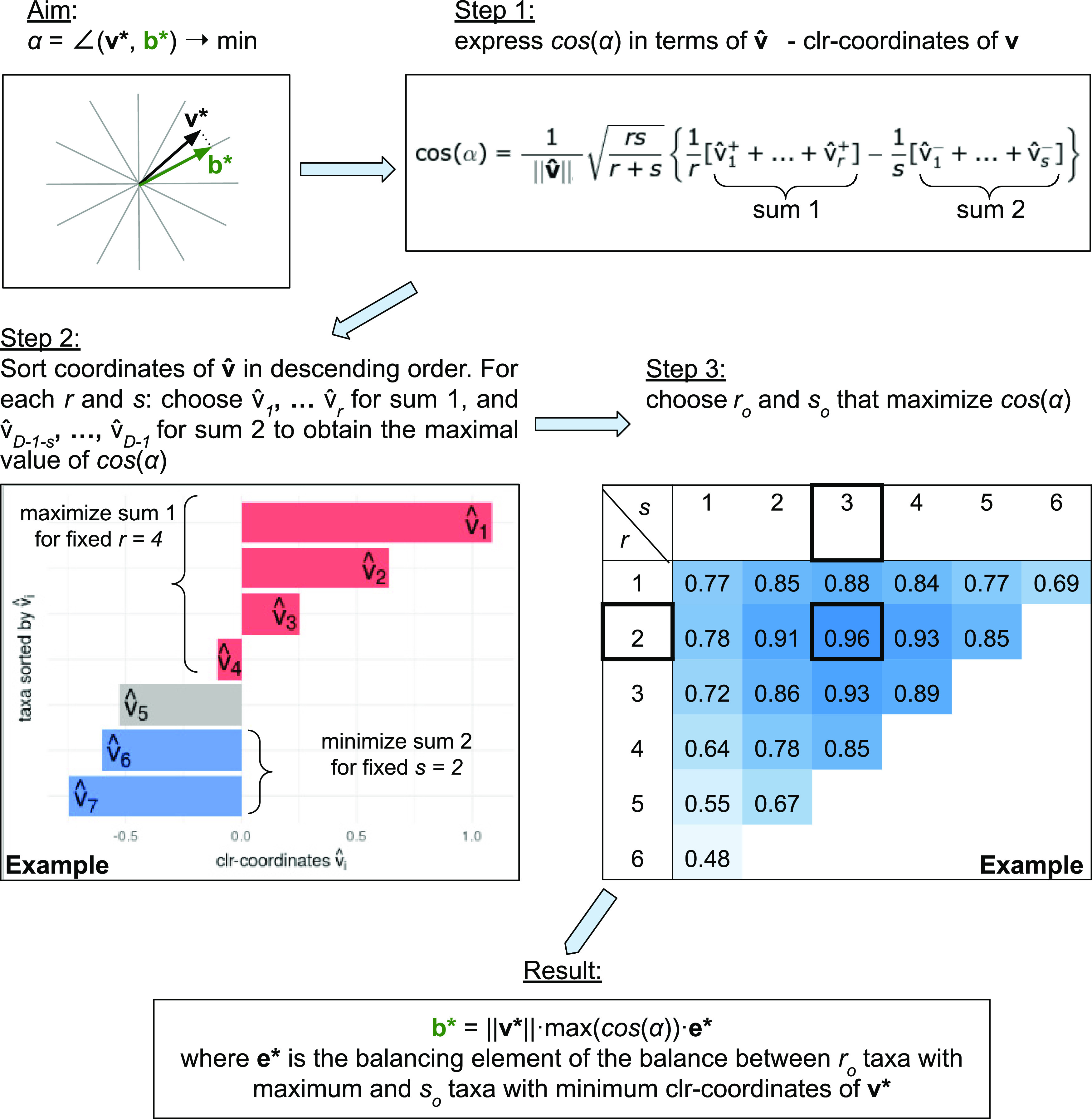
Diagram of algorithm A1 to find the nearest balance b* to an ILR vector v*.

Figure 3 shows the main steps of the main algorithm A1, which provides the nearest balance b to a given microbiome composition shift v. In brief, the cosine between the ILR components of the shift v* and an arbitrary balance b* is proportional to the difference between the means of centered log-ratio (CLR) coordinates that correspond to the groups of taxa in the numerator and denominator of the balance (step 1 in Fig. 3; see the proof in Materials and Methods). The balance between taxa with *r* maximum and *s* minimum CLR components of microbiome shift v is the closest to the shift (step 2 in Fig. 3). This is the key step of the algorithm, as it allows avoiding a grid search through all balances between fixed-sized groups. A search through all possible *r* and *s* values provides the optimal balance (step 3 and results in Fig. 3).

Although the algorithm exactly finds the closest balance, in practice it has some limitations. Assume the microbiome change is indeed a single-balance change b* and the observed vector of microbiome change v* differs from this single-balance change b* due to technical noise. If the noise level is sufficiently high, the observed change v* may become closer to another balance b*′. In such cases, the nearest balance algorithm will return a wrong balance b*′.

**FIG 4:**
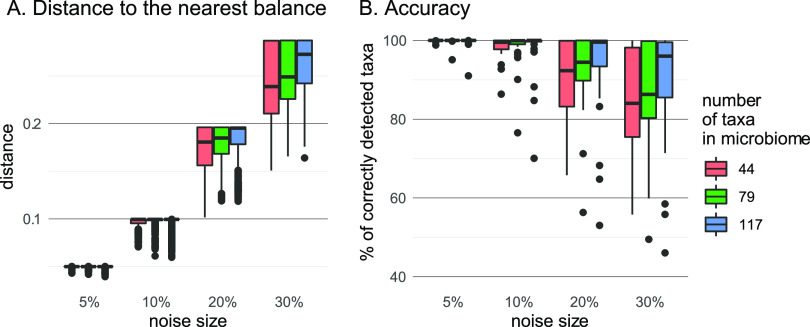
Stability of the nearest balance search to the noise in observations. (A) Distance from the disturbed vector to the nearest balance. If it is less than the noise size, it means that the disturbed balance becomes closer to a balance different from the original one. (B) Proportion of taxa correctly included in the numerator, denominator, or correctly excluded from the balance.

Figure 4 illustrates the effect of the noise level on the ability to reconstruct the correct balance on simulated data. For the simulation, we constructed three ILR systems of coordinates for a healthy human stool microbiome data set; the systems differ by the total number of taxa included in the analysis (see Materials and Methods). Each balancing element was perturbed in an orthogonal direction with a given noise size: 5, 10, 20, and 30% of its length. The procedure was repeated 20 times for each size of perturbation. The nearest balance was identified for each perturbed vector and compared to the initial vector. The accuracy of the method was measured as the proportion of taxa correctly included in the numerator or denominator or correctly excluded from the balance. Distance to the closest balance was compared to the *a priori* known size of the noise (i.e., distance to the disturbed balance).

As expected, the higher the noise, the more often the perturbed vector turns out to be closer not to the initial balancing element, but to some other element (Fig. 4A). The accuracy of detecting balance components decreases as the noise increases, too (Fig. 4B). The result depends on the total number of taxa in composition: in a higher-dimensional space larger noise is acceptable. For the tested values (from 44 to 117 taxa), noise that is ≤10% of the initial balance length leads to a rather low (<10%) proportion of mistakes.

### Single-balance regression analysis.

Multivariate linear regression in an ILR space is a promising tool for modeling microbiome composition dependence on various scalar factors, such as study group or age. Its coefficients are ILR vectors; they describe direction of changes associated with a unit change of a predictor. The nearest balance method may provide an interpretation of such alterations by changes in a single balance.

It turns out that such interpretation is optimal not only in terms of the difference between the coefficient and the balance but also in terms of the mean squared error of the prediction: the nearest balance is the least-squares estimate of the linear regression coefficient, given that it is restricted to be a balance vector (see Materials and Methods for the proof).

We compared the approach with several other compositional methods during cross-validation on synthetic and real data (see Fig. S1 in the supplemental material). The synthetic data set (1,000 samples per group 20 times randomly split into test and train subsets at 1:1 ratio) simulated a case-control study with a single-balance difference between the groups’ mean compositions. The real data set contains microbiomes of healthy subjects and subjects with Crohn’s disease (CD; 34 samples per group, 20 times randomly split into test and train subsets at a 1:4 ratio). The case-control design was selected for benchmarking, since it is simple and provides a relatively wide range of applicable compositional methods.

10.1128/msystems.00155-22.4 FIG S1 Scheme of the cross-validation for the single-balance regression problem and the classification problem. For *nb_lm*, *nb_lda*, and *nb_svm*, the discriminating balance was obtained with algorithm A1, and for the tree the discriminating balance was obtained with algorithm A3. *PDBA*, *ADBA*, and *hclust* provide the tree as a solution; the discriminating balance was chosen as the one whose variance is best explained by the group. The *selbal* method provides a discriminating balance; a random tree containing it was constructed for comparison. Download FIG S1, EPS file, 0.5 MB. Copyright © 2022 Odintsova et al. 2022 Odintsova et al. https://creativecommons.org/licenses/by/4.0/ This content is distributed under the terms of the Creative Commons Attribution 4.0 International license.

Since none of the existing compositional methods was intended exactly for interpreting linear regression coefficients, we adopted them, respectively. The *balance* R package (9) allows construction of an ILR coordinate system based on differences in microbiome composition between two groups in several ways: *PDBA* (the most differentiating balances include large number of taxa), *ADBA* (the most differentiating balances include small number of taxa), and *hclust* (clustering taxa as it is done in *gneiss* [10]). In addition, we applied the *selbal* algorithm and constructed a random ILR system of coordinates containing the identified balance. For each of the ILR systems, we selected the coordinate with the most explainable by the case-control factor variance. The regression parameters were optimized to describe the difference between groups by change in this single coordinate in the way to provide minimum squared error (see Materials and Methods). We compared the methods by the proportion of explained variance, MANOVA (or PERMANOVA if the sample size was too small) *P* values for all coordinates except for the main balance and the proportion of correctly detected taxa for the simulation (as described in the previous section).

**FIG 5:**
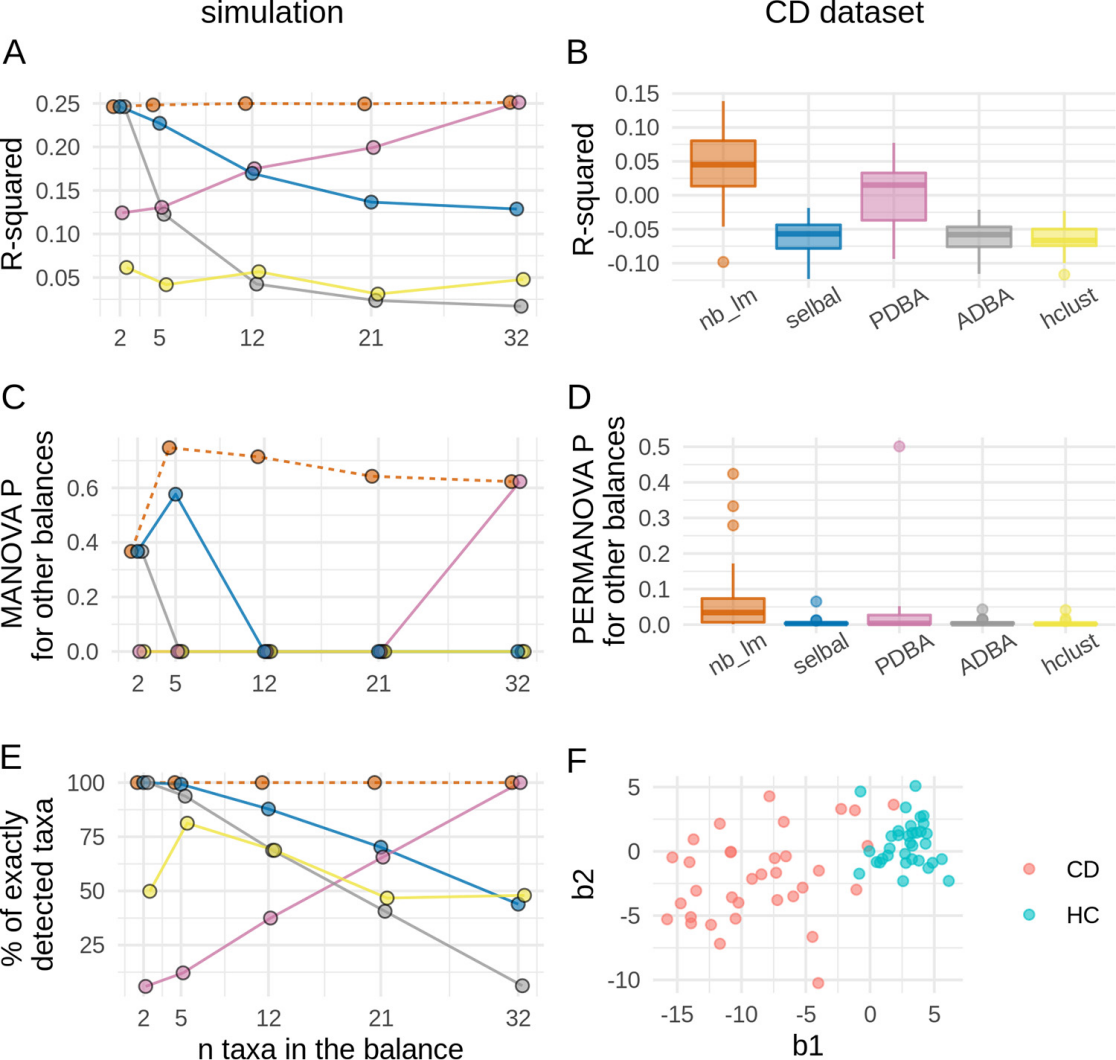
Results of the cross-validation for the regression problem. The left column illustrates results for the simulation and the right column shows those for the Crohn’s disease study (CD, *n* = 68). For the simulation, the mean values through all iterations of cross-validation are plotted; horizontal jitter is added to make points distinguishable. Boxplots are used to illustrate the results on the CD data set. (A and B) Proportions of explained variance on the test set. (C and D) *P* values for the MANOVA (for simulated data) or PERMANOVA (for CD data) analyses performed on all balances except for the main on the test set; if it is high (>0.05), it means that the main balance incorporates all the differences between two groups. (E) Proportion of taxa that were correctly included in the balance or excluded from it. (F) CD data set in coordinates obtained by *nb_lm* on the whole sample.

The proportion of explained variance [the R^2^ value was measured as 1 – (the mean squared error/the total variance)] is negatively related to the mean squared error; thus, it directly reflects the optimization criterion of linear regression. The nearest balance method guarantees the highest value on the train subset. Figure 5 shows the results on the test subset. The *nb_lm* method showed best results both on simulated and real data sets (Fig. 5A and B). The results of the simulation show that the predictive power of other methods depends on the number of taxa included in the discriminating balance. *ADBA* and *PDBA* coordinates incorporate assumptions about this number and thus work reasonably well if they are satisfied and less accurate otherwise. R-squared for the *selbal* prediction decreased with increasing balance size. This is partly caused by an artificial restriction: by default, the algorithm searches for balances of between ≤20 taxa. Besides the fact that this setting is usually used in practice, another reason to use it was that the increase of the balance size considerably slows down the cross-validation. The *hclust* method showed a relatively low R-squared independently from the number of taxa included in the balance.

The *nb_lm* method was also best for determining the members of the balance (Fig. 5E) on the simulated data. The precision of other balances depended on the balance size.

The MANOVA test was used to test the hypothesis that there is no difference in balances orthogonal to the main one. High *P* values (>0.05) mean that all differences between two groups are incorporated in a single balance. Figure 5C shows that nearest balance was the only method that showed high *P* values independently of the balance size. The sample size of the CD data set was too small to evaluate MANOVA; thus, a nonparametric analogue—PERMANOVA—was applied (Fig. 5D). None of the methods provided a balance incorporating all of the differences between groups in all cross-validation iterations; the *nb_lm* method yielded higher *P* values.

This difference in MANOVA *P* values on simulated and PERMANOVA on the real data may be explained by the difference in the noise size. The noise observed for the shift may be estimated as the standard deviation (SD) of the coefficient of linear regression. For the simulated data, the noise was about 4% of the shift between groups, while for the CD data set it was 25%. As discussed above, the latter value is rather high.

The ILR coordinates constructed by *nb_lm* are useful for the visualization of the regression results (Fig. 5F). For CD data, healthy and control subjects are clearly distinct.

### Classification.

**FIG 6:**
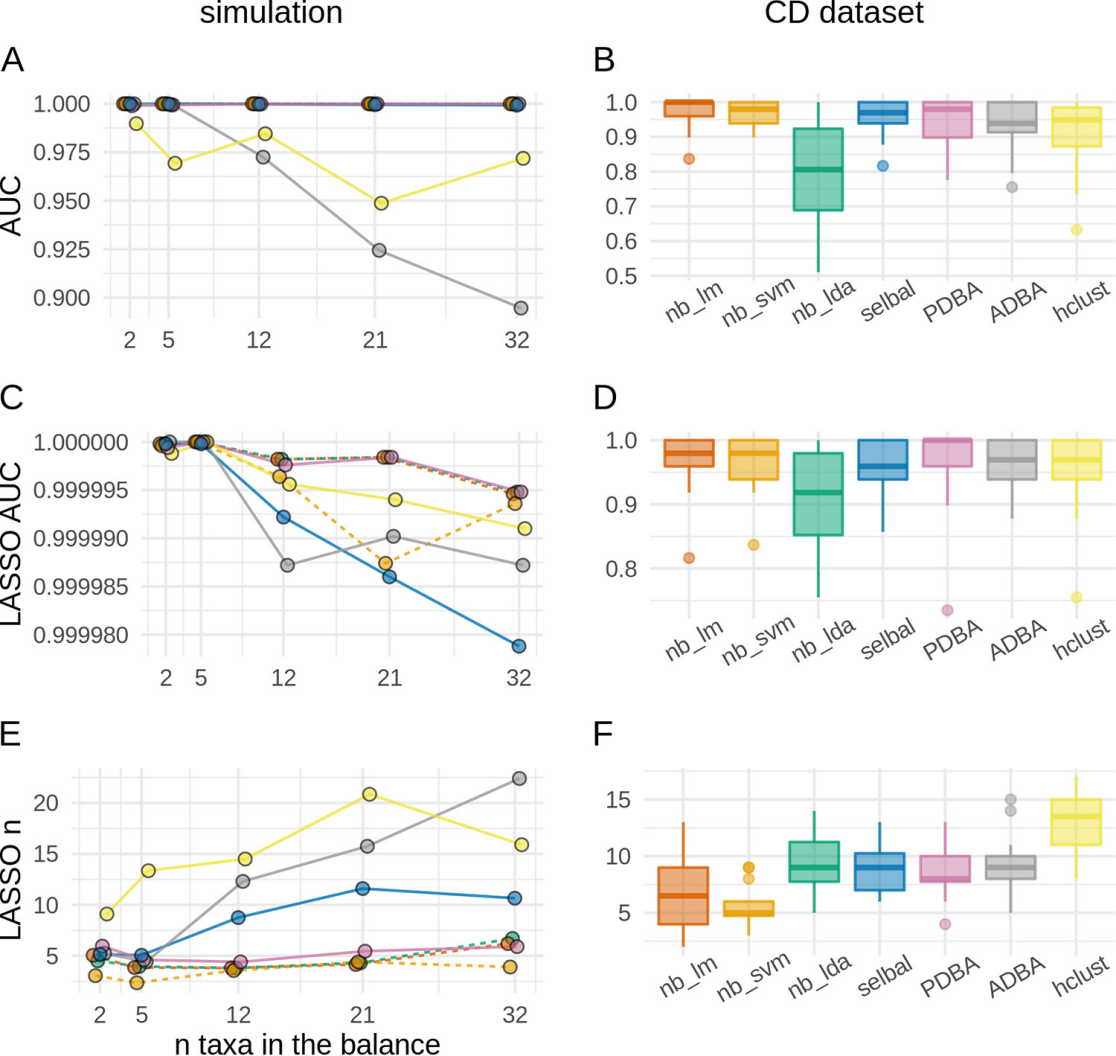
Results of the cross-validation for the classification problem. The left column illustrates results for the simulation, and the right shows the results for the Crohn’s disease study (CD). For the simulation, the mean values through all iterations of cross-validation are plotted. Boxplots are used to illustrate the results on the CD data set. Horizontal jitter is added to the plot to make points more distinguished. (A and B) AUC of classification by a single balance obtained by various methods. (C and D) AUC of classification by a LASSO regression trained in coordinate systems obtained by various methods. (E and F) Number of balances selected by the LASSO regression classifiers.

The same simulated and real data sets were used to benchmark classification by a single balance (see the description for Fig. S1 and above). In addition to the methods described above, we performed the nearest balance combined with LDA (*nb_lda*) and SVM (*nb_svm*). The values for the area under the receiver operating characteristic curve (AUC) for simulated data were high for all methods (Fig. 6A). As for the CD data set, all methods except *nb_lda* provided comparable AUC values (Fig. 6B).

In addition, we compared the ILR coordinate systems provided by these methods. For this purpose, the LASSO regression was fitted. A good coordinate choice should result in a high AUC value and a low number of balances remaining in the model. All methods except for *nb_lda* yielded high AUC values on both simulated and real data sets (Fig. 6C and D). The lowest numbers of balances were produced by the *nb_lm*, *nb_svm*, *nb_lda*, and *PDBA* methods on simulated data and by *nb_lm* and *nb_svm* on real data (Fig. 6E and F).

### Principal balance analysis.

We compared application of algorithm 3 to PCA interpretation (*nb_pca*) with several methods of principal balance analysis: “*cluster*” and “*constrained*” implemented in the *pb_basis*() function of R package *coda.base* (8) and anti-principal balance analysis (*ABA*) from the *balance* package on the CD data set. The proportions of variation explained by the first two balances by each method are presented in Table 1. *nb_pca* and *constrained* provided the best variance explained by the first coordinate and, by the first two coordinates, *nb_pca* was slightly better.

**TABLE 1:** Proportion of variance explained by the first two balances (b_1_ and b_2_) obtained by several principal balance analysis methods applied to the Crohn’s disease microbiome data set^*a*^

Method	b_1_ (%)	b_2_ (%)	Sum (%)
*nb_pca*	25.00	9.54	34.54
*constrained*	24.71	9.54	34.25
*cluster*	20.52	9.96	30.48
*ABA*	8.62	7.49	16.11

a The following notations are used: *nb_pca*, nearest balances approach; *constrained* and *cluster*, methods implemented in R package *coda.base*; *ABA*, anti-principal balance analysis implemented in the R package *balance.*

### Example: microbiome composition shift associated with Crohn’s disease.

To illustrate the biological relevance of our method, we applied single-balance regression to investigate the microbiome shift in patients with Crohn’s disease (CD). Comparison data sets from two studies with distinct populations (and likely varying by the experimental protocols) allowed us to evaluate the reproducibility potential of the method.

Data Set 1 is the one used for cross-validation above. Data Set 2 contains microbiome composition data on healthy subjects and patients obtained from reference 12. For consistency, we considered proportions of only those 38 taxa remaining after filtering Data Set 2. Below, the single-balance regression trained on Data Set 1 is denoted model 1, while the one corresponding to the Data Set 2 is denoted model 2.

**FIG 7:**
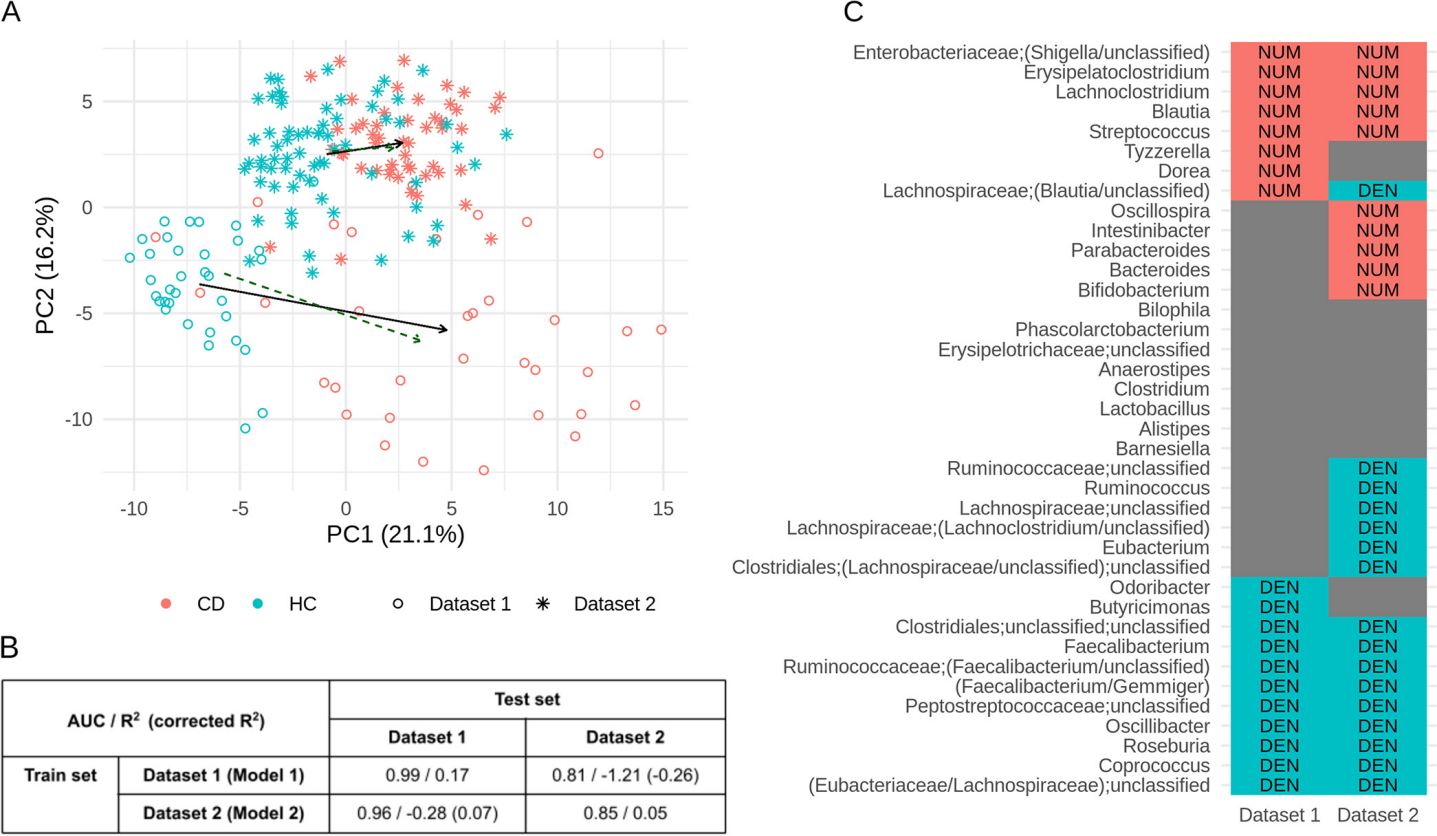
Single-balance regression applied to two studies comparing gut microbiomes of healthy subjects and patients with Crohn’s disease. (A) Principal-component analysis (in an ILR space). Arrows indicate predictions of multivariate linear regression (solid black) and single-balance linear regression (dashed green). (B) Results of single-balance linear regressions trained and tested on two data sets. AUC, R-squared, and corrected (for systematic shift between data sets) R-squared results are shown. (C) Members of balances nearest to the CD-associated microbiome shifts for each data set. Red NUM, taxon is in the numerator; blue DEN, in the denominator.

Figure 7A shows clear differences in ILR space between the two studies, as well as between CD patients (CD) and healthy controls (HC) within each study. The difference between HC and CD samples is clearer in Data Set 1. This fact is supported by the multivariate linear regression analysis: Data Set 2 data set are noisier (36% versus 24%), and the size of the shift is three times smaller (4.1 versus 12.5, Aitchison norm of the shift). As a result, the quality of the single-balance regression model is higher for the less noisy Data Set 1: model 1 has higher R-squared (0.17 versus 0.05, Fig. 7B) and AUC (0.99 versus 0.85) values than model 2. The shift is statistically significant in both data sets (*P* = 8E–07 in Data Set 1 and *P* = 6E–08 in Data Set 2, MANOVA for all ILR coordinates). Both models provide MANOVA *P* < 0.05 for the orthogonal to main balance subspace, suggesting that the difference between CD and HC microbiomes is only partially described by a single balance in both data sets. However, the impact of the main balance in this difference is considerable: 83.6% for model 1 and 86.8% for model 2.

Members of the balances obtained via single-balance regression are shown in Fig. 7C. Taxa with an ambiguous classification at the genus level were marked using “/” and augmented with their family name [e.g., “*Lachnospiraceae;(Blautia/unclassified)*”]. Each balance splits taxa into three categories: numerator of the balance, its denominator and taxa excluded from the balance. Only 22 of 38 taxa (57%) are equivalently categorized by the two models. However, the balances are almost noncontradictory except for one genus-level taxon—“*Lachnospiraceae;(Blautia/unclassified)*”—that is positively associated with the disease in model 1 and negatively associated with the disease in model 2. The noisier Data Set 2 yielded a balance that included a higher number of taxa.

Next, model 1 was cross-tested on Data Set 2, and model 2 was cross-tested on Data Set 1. The predictive power of single-balance regression itself was low (R-squared is negative). It can be in part explained by the systematic shift: R-squared is 0.07 for model 2 tested on the Data Set 1 corrected for this shift. Another source of error is the difference in effect size of the CD-associated shift between Data Sets 1 and 2: as discussed above, it was three times larger in Data Set 2.

The direction of the shift itself had a considerably high predictive power for both models. Model 1 has an AUC of 0.81 for Data Set 2 (versus AUC = 0.85 for model 1), while model 2 has an AUC of 0.96 for Data Set 1 (versus AUC = 0.99 for model 1).

As for the members of balances, the two denominators mostly include genera from the *Clostridiales* order, many of which are known as prominent producers of butyrate, a short-chain fatty acid crucial for gut health. Depletion of butyrate production potential is a hallmark of the CD microbiome (13, 14). The list of butyrate producers includes *Faecalibacterium*, *Roseburia*, and *Coprococcus* (15, 16), also previously reported to be negatively associated with CD in noncompositional systematic meta-analyses (14, 17). The mechanism of *Faecalibacterium* participation in intestinal inflammation, particularly during CD, is not limited to butyrate production; the bacteria can produce other microbial anti-inflammatory molecules (18). *Roseburia* is a gut microorganism found to be depleted not only in the microbiome of CD patients but also in healthy subjects with high genetic risk of inflammatory bowel disease (IBD) (19).

On the other hand, the intersection of the two numerators includes taxa for which strong positive associations with the CD and/or other gastrointestinal disorders have been demonstrated in the literature, along with possible mechanisms. For example, the “*Enterobacteriaceae;(Shigella/unclassified)*” taxon is related to Escherichia coli, the species often enriched in the guts of CD patients. Its adherent-invasive pathotypes (AIEC) are known to be able to invade epithelial cells in the disease, and a wide genomic diversity of CD-associated E. coli strains has been reported as well (13). Other taxa included in the numerator have been implicated in pathologies. For example, *Erysipelatoclostridium*, opportunistic clostridia linked to diseases that include metabolic disorders, gout (20), and IBD (21); in addition, another numerator member, *Lachnoclostridium*, was linked to colorectal adenoma (22) and atherosclerosis (23).

## DISCUSSION

We propose a *post hoc* method to interpret compositional analysis results: the microbiome changes are optimally approximated by a shift in a single balance between two groups of taxa (i.e., the nearest balance). It is compatible with any statistical method that yields a vector in ILR coordinates, such as coefficients of a mixed-effect model, a vector normal to a plane obtained via a linear classifier, PCA axis, or a vector of difference between two compositions.

Such an approach does not guarantee optimality in terms of the initial problem optimization criterion. Thus, we explored the accuracy of several applications—regression, classification problems, and principal balance analysis—with analytical treatment and/or cross-validation.

The approach provides the least-squared error for the single-balance regression problem, i.e., for the search for single-balance changes in a microbiome associated with a factor possibly adjusted for several covariates. Three simple examples of such analysis are the approximation of case-control differences, mean changes in the microbiome of a group of subjects, or a single subject’s microbiome shift by a single-balance change. As for the third case, to our knowledge, the nearest balance approach is the first compositional method to solve the problem.

The method is comparable to other compositional approaches for the classification problem and principal balance analysis. Its advantage is lack of a prior on the number of components in the resulting balances and rather low computational complexity (except for algorithm A3).

Application to the gut microbiome in Crohn’s disease showed that the method provides biologically meaningful and reproducible results. Two models trained on two data sets from distinct populations provided considerably overlapping balances. These balances reflect a trade-off between commensal butyrate-producing taxa and taxa linked to negative effects on gut health. The single-balance models showed low predictive power in cross-testing (due to differences in populations), but the balances themselves allowed good performance for classifying subjects into healthy ones and patients, suggesting the biological relevance of the results.

The presented method shares the limitations of any compositional analysis: the ILR transform is not compatible with zero abundance values, and the total sum of counts per sample (and thus the information about the precision of the measurement) is lost while converting to proportions. In addition, the amalgamation of taxa into groups presents a separate issue that can influence the results (24). Another limitation concerns the assumption that the nearest balance reveals the biological interpretation of the vector. It is violated if the approximated vector is a superposition of changes in several, possibly nonorthogonal directions. This problem may be partially solved by a careful choice of covariates in the model.

The implementation needs further improvement. First, if several balancing elements are equally close to the approximated vector, only one of them is detected. As a consequence, the search for the second balance orthogonal to the first one, as well as the search for the nearest balance tree, may be suboptimal. Second, the search for two orthogonal balances is based on the assumption that they correspond to the nodes of the same binary tree; other possibilities have not yet been explored.

To summarize these findings, the nearest balance is a compositional method providing optimal solutions for single-balance regression and extending the opportunities for classification, principal balance analysis, and other methods of treating compositional data.

## MATERIALS AND METHODS

### Basic definitions.

The detailed theory of compositional analysis may be found in reference 3. Here, we list several definitions and properties essential for understanding this method.

Composition is a vector that contains the information about relative proportions of components, while the total sum is of no concern. Normalization of a composition to a certain total sum is called a closure operation.

An ILR transform should be applied for the correct treatment of such data. It converts data to a Euclidean space where operations with compositions are calculated as common operations with vectors. The ILR transform is not unique: its particular form yields a choice of coordinates in the space; any orthogonal system is appropriate for the analysis provided its origin corresponds to the composition with equal proportions of all parts. One of the ways to define such a transform is based on the construction of a binary tree with features as leaves (in case of microbiome data the features are microbial taxa). ILR coordinates of a vector v are calculated as balances that correspond to each node *i* of the tree (Fig. 1C) in the following way:

$$\mathrm{ilr}\left(\text{v}\right)\;={\text{v}}^{*}=\;\left({{\text{v}}^{*}}_{1},\;{{\text{v}}^{*}}_{2},\;\ldots \;{{\text{v}}^{*}}_{D-1}\right)$$

$${{\text{v}}^{*}}_{i\;}=\sqrt{\frac{\mathrm{rs}}{r\;+s}}\text{ln}\frac{{\text{g}}_{m\;}({\text{Num}}_{i\;})\;}{{\text{g}}_{m\;}({\text{Den}}_{i\;})}$$

where Num*_i_* and Den*_i_* denote two sets of leaves that correspond to one of the child branches of node *i*, g*_m_* denotes the geometric mean of relative abundances of taxa that correspond to these leaves and *D* is the dimension of the initial space of proportions (i.e., the total number of taxa). The balances may be considered independently of the ILR system construction, since they characterize the relations between groups of taxa in a composition. The change of a balance defines a direction in the Euclidean space; a unit vector in this direction is called a balancing element.

The centered log-ratio (CLR) transform (which is the reverse *softmax* function) is another transform that preserves all operations with compositional vectors and converts data to a Euclidean space. Components of the transformed vector are calculated as follows:

$$\mathrm{clr}\left(\text{v}\right)=\hat{\text{v}}=\left({\hat{\text{v}}}_{1},{\;\hat{\text{v}}}_{2},\;\ldots ,{\;\hat{\text{v}}}_{D}\right)$$

$${\hat{\text{v}}}_{i}=\text{ln}\left({\text{v}}_{i}\right)-\sum_{j=1}^{D}\text{ln}\left({\text{v}}_{j}\right)/D$$

Unlike the ILR transform, CLR transform does not reduce the dimension of vectors, leaving the problems of components’ interdependence unresolved; thus, this method is appropriate for statistical treatment of distances between compositions, but not for component-wise analysis.

The ILR and CLR coordinates are related in the following way:

$${\text{v}}^{*}=\hat{\text{v}}\Psi^{\text{T}}$$

$$\hat{\text{v}}={\text{v}}^{*}\Psi$$

where the superscript “T” denotes transposition, Ψ is a (*D *– 1) *× D* matrix with CLR coordinates of the basis vectors of the ILR system in the rows. Each basis vector represents a balancing element e* **=** (e*_1_, …, e*_K-1_). Its CLR coordinates ê = (ê*_1_*, …, ê_*D*−1_) are equal to:

$${\text{a}}_{+}\left(r,s\right)=\frac{1}{r}\sqrt{\frac{\mathrm{rs}}{r+s}}$$

for *r* components corresponding to the numerator of e*,

$${\text{a}}_{-}\left(r,s\right)=-\frac{1}{s}\sqrt{\frac{\mathrm{rs}}{r+s}}$$

for *s* components corresponding to its denominator and 0 for all others.

Since both CLR and ILR transforms preserve the distance and inner product, the following equalities are valid for any two compositions v and w:

$$\langle \hat{\text{v}},\hat{w}\rangle =\langle {\text{v}}^{*}\text{,}{\text{w}}^{*}\rangle$$

$$\left\|\hat{\text{v}}\right\|\;=\;\left\|{\text{v}}^{*}\right\|.$$

where <,> denotes the inner product, and ‖.‖ denote the Euclidean norm in CLR or ILR space.

### Suggested algorithms. (i) Algorithm A1 (ILR vector → the nearest balance).

Here, the nearest balancing element to a vector v* is understood as the balancing element that includes the minimum angle to v* in an ILR space. Since the balancing element is a vector of unit length, the cosine between a balancing element e* and vector v* may be calculated as follows:

$$\text{cos}\left(\alpha \right)=\frac{\langle {\text{v}}^{*}\text{,}{\text{e}}^{*}\rangle }{\left\|{\text{v}}^{*}\right\|}\text{=}\frac{\langle \hat{\text{v}}\text{,}\hat{e}\rangle }{\left\|\hat{\text{v}}\right\|}$$

It reaches its maximum value when 〈v*,e*〉_*ilr*_ is maximum. The coordinates of v̂ are calculated as v*Ψ and CLR coordinates ê of element e take one of the values {0; *a_+_*(*r*,*s*); *a*_–_(*r*,*s*)}. The cosine of the angle between the balancing element and the vector is described by the formula:

$$\text{cos}\left(\alpha \right)=\frac{1}{\left\|\hat{\text{v}}\right\|}\sqrt{\frac{rs}{r+s}}\left\{\frac{1}{r}\left[{\hat{\text{v}}}_{1}^{+}+\;\ldots \;+{\hat{\text{v}}}_{r}^{+}\right]\;-\;\frac{1}{s}\left[{\hat{\text{v}}}_{1}^{-}+\;\ldots \;+{\hat{\text{v}}}_{s}^{-}\right]\right\}$$

where ${{\hat{\text{v}}}^{+}}_{i}$ values are *r* components corresponding to the taxa in the numerator of e*, and ${\hat{\text{v}}}_{i}^{-}$ values are *s* components related to its denominator. For a fixed *r* and *s*, the expression reaches the maximum value when the sum $\left[{\hat{\text{v}}}_{1}^{+}+\;\ldots \;+{\hat{\text{v}}}_{r}^{+}\right]\;$is the maximum and $\left[{\hat{\text{v}}}_{1}^{-}+\;\ldots \;+{\hat{\text{v}}}_{s}^{-}\right]$ is the minimum. For fixed *r* and *s*, the maximum sum of *r* components is provided by summing the *r* maximum components of vector v̂, and the minimum sum of *s* components is provided by summing its *s* minimum components. If the components of v̂ are descendingly ordered, the minimum cos(α) equals:

$$\text{cos}\left(\alpha \right)=\frac{1}{\left\|\hat{\text{v}}\right\|}\sqrt{\frac{rs}{r+s}}\left\{\frac{1}{r}\left[{\hat{\text{v}}}_{1}\;+\;\ldots \;+\;{\hat{\text{v}}}_{s}\right]\;-\;\frac{1}{s}\left[{\hat{\text{v}}}_{D+1-s}\;+\;\ldots \;+\;{\hat{\text{v}}}_{D}\right]\right\}.$$

Thus, for each *r* and *s*, the nearest balancing element to v* may be obtained by uniting *r* taxa with maximum CLR coordinates in one group and *s* taxa with minimum coordinates in the other group. Now, a simple search through all possible values of *r* and *s* leads to the optimal solution.

Vector v* may be approximated by its projection on the nearest balancing element. We call this projection b*, the nearest balance. It has the least possible angle to v* and is the closest to it among all balance vectors colinear to e*, thus b* satisfies the equality:

$${\text{b}}^{*}\;=\;\underset{\beta \in B^{D\;-\;1}}{\text{argmin}}{\left\|\beta \;-\;{\text{v}}^{*}\right\|}^{2},$$

where *B^D^*^-1^ denotes a set of all balance vectors in ℝ*^D^*^-1^ (the ILR space).

The impact of b* to the total vector v* is estimated as ‖b*‖^2^/‖v*‖^2^: the nearest balancing element may be considered as a basis vector of an ILR system, and this definition gives a unit total sum for the impacts of all axes.

### (ii) Algorithm A2 (two ILR vectors → two orthogonal balances).

Let us find balancing element e*_2_ that is the closest to an ILR-vector w* among all balancing elements that may be observed in one and the same binary tree with e*_1_ (and thus orthogonal to it [6]). Vector w* can be equal to v*, if the aim is a more exact approximation, but it can be a new vector as well if the aim is, for example, an approximation of the PCA axes.

The constraint of e*_1_ and e*_2_ being from the same binary tree implies that one of the following conditions must be satisfied (see Fig. S2): (case A) node e*_2_ is inside one of the child branches of node e*_1_; (case B) node e*_1_ is inside one of the child branches of node e*_2_; and (case C) child branches of nodes e*_1_ and e*_2_ have no common leaves (the nodes are named after the balancing elements).

10.1128/msystems.00155-22.5 FIG S2 Cases of relations between e*_1_ and e*_2_ considered in algorithm 2: an example. Nodes A_1_ and A_2_ are inside one of the e*_1_ child branches. Nodes B include all leaves of e*_1_ inside one of its child branches. Nodes C have no common child branches with e*_1_. Download FIG S2, EPS file, 0.02 MB. Copyright © 2022 Odintsova et al. 2022 Odintsova et al. https://creativecommons.org/licenses/by/4.0/ This content is distributed under the terms of the Creative Commons Attribution 4.0 International license.

In cases A and C, nonzero CLR coordinates of e*_2_ are related only for a certain group of taxa: either the numerator or denominator of the first balance in case A and all other taxa in case C. Reduction to a subspace of all nonzero e*_2_ components preserves the inner product with w*. Thus, the best balancing element among each of these groups may be found in the same manner that is described in the previous section: w* coordinates that correspond to certain subspace are ordered decreasingly, and inner products of e*_2_ and w* are calculated for all variants of *r* and *s* and then compared.

In case B, the procedure is slightly more complicated, since all components related to e*_1_ should be considered together. For example, an algorithm to search for the balance containing all taxa related to e*_1_ in its numerator includes the following steps: (i) order decreasingly components of ŵ that were not included in the first balance; (ii) place the remaining taxa at the beginning of the list; (iii) calculate the inner product with ŵ for each *r* and *s* similarly as described for the first balance and v̂, with the only difference that only values of *r* greater than the number of taxa in the first balance should be considered; and (iv) choose the optimal *r* and *s*. The first two steps ensure that the condition B is satisfied; the second and third steps provide maximum inner product for the given *r* and *s*. The algorithm of searching for the balance that contains all taxa from the first balance in its denominator is similar; the only difference is that the components that correspond to the first balance should be placed at the end of the list.

Text S1 shows that b*_2_ is the least-squares approximation of w* by its superposition with a given orthogonal balance b*_1_ among all orthogonal to b*_1_ balances.

10.1128/msystems.00155-22.1 TEXT S1 Proof of a statement about superposition of the balances obtained by algorithm A2. Download Text S1, DOCX file, 0.01 MB. Copyright © 2022 Odintsova et al. 2022 Odintsova et al. https://creativecommons.org/licenses/by/4.0/ This content is distributed under the terms of the Creative Commons Attribution 4.0 International license.

### (iii) Algorithm A3 (an ILR vector → the nearest balance tree).

The aim of this algorithm is to construct an ILR system of coordinates with basis {e*_1_, e*_2_, …, e*_D__-1_}, such that e*_1_ is the nearest one to vector v*, the e*_2_ being the nearest to v* among all elements orthogonal to e*_1_, the third element being the nearest to v* among all balances orthogonal to e*_1_ and e*_2_ and so on. This algorithm is constructed recursively similar to algorithm A2: the taxa are stepwise split into groups to provide orthogonality of balances, on each step the cosine is maximized for fixed *r* and *s* (see Fig. S3), and the optimal values are chosen. The main difficulty is in the choice of elements that maximize the cosine for a given *r* and *s*. Similarly to A2, some elements are united in a branch on previous steps and should be included or excluded from the numerator or denominator of the new balance together. In the case of A2 the number of groupings is 1, but in this algorithm it may be larger. To resolve this difficulty, at each step we substitute components of v̂ that are included in the same branch by their means and obtain a new vector v̂′. Next, we assign weights to its components: “1” for the ones that came from the original vector v̂ and the number of substituted components for the others. The sum of v̂ components equals the weighted sum of v̂′ components. A simple search through all combinations of weights summing to *r* or *s* gives the maximal cosine with v̂ among all balances orthogonal to the previously found ones (for fixed *r* and *s*). The *partitions* package (25) is used to implement the search for possible combinations of weights. Figure S3 provides a detailed algorithm scheme, and Text S3 illustrates this with a simple example.

10.1128/msystems.00155-22.3 TEXT S3 An example of constructing a binary tree according to algorithm A3. Download Text S3, DOCX file, 0.02 MB. Copyright © 2022 Odintsova et al. 2022 Odintsova et al. https://creativecommons.org/licenses/by/4.0/ This content is distributed under the terms of the Creative Commons Attribution 4.0 International license.

10.1128/msystems.00155-22.6 FIG S3 Schematic representation of the algorithm A3, which constructs a nearest-balance tree. Labels s1, …, s9 denote the steps referred to in the tree construction example (see Text S3). Download FIG S3, PDF file, 0.05 MB. Copyright © 2022 Odintsova et al. 2022 Odintsova et al. https://creativecommons.org/licenses/by/4.0/ This content is distributed under the terms of the Creative Commons Attribution 4.0 International license.

### Single-balance linear regression.

Consider a linear dependence between ILR coordinates of a *D*-part composition y* (e.g., microbiome composition consisting of *D* taxa in various proportions represented by a vector with (*D*-1) ILR components), a univariate predictor x (e.g., a dosage of a medicine or a binary variable representing presence of a certain disease) and several univariate covariates z_1_, …, z*_K_* (e.g., *K *=* *2, z_1_ is age, and z_2_ is an integer value representing gender of a participant):

$${\text{y}}^{*}={\text{xv}}^{*}\;+\;{\text{z}}_{1}{a^{*}}_{1}{\;+\;\ldots \;+\;\text{z}}_{K}{a^{*}}_{K}.$$

Coefficients of this linear dependence v* and a*_1_, …, a*_K_ are (*D*-1)-dimensional vectors in the ILR space. Each of them represents an ILR shift of composition y* associated with a unit change of a predictor or a covariate (e.g., presence of a disease, a unit change of a medicine dosage, or a unit change in age). These coefficients are often an object of interest. In practice they may be estimated basing on known values of the composition y*, predictor x, and covariates z_1_, …, z*_K_* in a sample and assuming a random measurement error in observations.

The ordinary multivariate linear regression model assumes that composition of each sample in ILR space is a random vector from a multivariate normal distribution with a mean being linear dependent on the predictor and the covariates, i.e., for each *i *=* *1, …, *N*:

$${y^{*}}_{i}\;\sim \;\text{N}({\text{x}}_{i}{{\text{v}}^{*}\;+\;\text{z}}_{1i}{a^{*}}_{1}{\;+\;\ldots \;+\;\text{z}}_{\mathrm{Ki}}{a^{*}}_{K}\text{,}\;\sigma^{2}I\text{)}$$

or in matrix representation:

$${y^{*}}_{i}\;\sim \;N({\text{x}}_{i}{\text{v}}^{*}\;+\;A^{*}{\text{z}}_{i}\text{,}\;\sigma^{2}I\text{)}$$

where y*_i_ is an (*D*-1)-dimensional ILR vector representing microbial composition of the *i*th sample, x*_i_* is the predictor’s value for the sample, and z*_i_* = [z_1_*_i_*, …, z*_Ki_*]^T^ is the vector of covariates’ values for it, *σ*^2^ is the variance parameter for the model, *N* is the sample size, and N(···,···) denotes a multivariate normal distribution with a given mean vector and covariance matrix.

The suggested single-balance linear regression model assumes a similar distribution but restricts the predictor’s coefficient to be a balance vector. It means that a unit change of the predictor is associated with a change in a single balance between two groups of parts (taxa):

$${{\text{y}}^{*}}_{i}\;\sim \;N({\text{x}}_{i}{\text{b}}^{*}\;+\;{A^{*}}_{\text{sb}}{\text{z}}_{i}\text{,}\;\sigma_{\mathrm{sb}}^{2}I\text{)},$$

where b* is a single-balance shift associated with the predictor x, A*_sb_ is the matrix of covariates’ coefficients, and $\sigma_{\mathrm{sb}}^{2}$ is the variance parameter for the model.

Below we use matrix representation of the sample’s characteristics: Y* = [y*_1_,  …,  y*_N_] = [y*_ji_] is a (*D*-1) × *N* matrix with observed ILR coordinates of the compositions, Z = [z_1_, …, z*_N_*] = [z*_ki_*] is a *K *×* N* matrix of covariates values, X = [x_1_, …, x*_N_*]*^T^* is a 1×*N* matrix of the predictor’s values, *j *=* *1, …, (*D*-1) is an index of ILR coordinate, and *k *=* *1, …, *K* is an index of a covariate. Using these notifications, we propose a statement about relationship between least-squares solutions of an ordinary multivariate linear regression model and a single-balance regression model.

*Theorem*. Let v*_ls_ be the least-squares estimate of coefficient v*, β_ls_ be nearest to the v*_ls_ balance vector, and *A_ls_* = (Y*– β_ls_X^T^)Z*^T^* (ZZ^T^)^–1^. Then, [β_ls_, *A_ls_*] is the least-squares estimate of the single-balance regression coefficients [b*, A*_sb_].

The proof of the theorem in the most common case is provided in Text S2. Below, two simple cases are described: the approximation of the mean shift by a single-balance change and a single-balance model with a single predictor.

10.1128/msystems.00155-22.2 TEXT S2 Proof of nearest balance being the least squares estimate of single-balance regression coefficient in the most common case. Download Text S2, DOCX file, 0.02 MB. Copyright © 2022 Odintsova et al. 2022 Odintsova et al. https://creativecommons.org/licenses/by/4.0/ This content is distributed under the terms of the Creative Commons Attribution 4.0 International license.

It is worth noting that the least-squares solution for the single-balance model coincides with the maximum-likelihood solution for the same reason that it is true for the ordinary linear regression with diagonal covariance matrix.

### (i) Approximation of the mean shift.

If x_i_ is 1 for all samples and the covariates are absent, the linear regression and single-balance regression reduce to models:

$${{\text{v}}^{*}}_{i}\;\sim \;N({\text{v}}^{*}\text{,}\;\sigma^{2}I\text{)}$$

and

$${{\text{v}}^{*}}_{i}\;\sim \;N({\text{b}}^{*}\text{,}\;\sigma_{\mathrm{sb}}^{2}I\text{)}$$

The least-squares (and maximum likelihood) estimate of v* is the mean in the sample. The least-squares estimate of b* is a balance vector β_ls_ that satisfies the following:

$$\beta_{\mathrm{ls}}=\underset{\beta \in B^{D-1}}{\text{argmin}}\sum_{i=1}^{N}{\left\|{{\text{v}}^{*}}_{i}\;-\;\beta \right\|}^{2}.$$

Since the argmin is independent of multiplying the function by a positive number and adding a constant,

$$\beta_{\mathrm{ls}}=\underset{\beta \in B^{D-1}}{\text{argmin}}\sum_{i=1}^{N}{\left({{\text{v}}^{*}}_{i}\;-\;\beta \right)}^{T}\left({{\text{v}}^{*}}_{i}\;-\;\beta \right)=$$

$$=\underset{\beta \in B^{D-1}}{\text{argmin}}\sum_{i=1}^{N}\left({{\text{v}}^{*}}_{i}^{T}{{\text{v}}^{*}}_{i}\;-\;2\beta^{T}{{\text{v}}^{*}}_{i}\;+\;\beta^{T}\beta \right)=$$

$$=\underset{\beta \in B^{D\;-\;1}}{\text{argmin}}\left[N\beta^{T}\beta \;-\;2\beta^{T}\sum_{i=1}^{N}{{\text{v}}^{*}}_{i}\right]=$$

$$=\underset{\beta \in B^{D\;-\;1}}{\text{argmin}}\left[\beta^{T}\beta \;-\;2\beta^{T}\sum_{i=1}^{N}{{\text{v}}^{*}}_{i}/N\right]=$$

$$=\underset{\beta \in B^{D\;-\;1}}{\text{argmin}}{\left(\beta \;-\;\sum_{i=1}^{N}{{\text{v}}^{*}}_{i}/N\right)}^{T}\left(\beta \;-\;\sum_{i=1}^{N}{{\text{v}}^{*}}_{i}/N\right)=$$

$$=\underset{\beta \in B^{D-1}}{\text{argmin}}{\left\|\beta \;-\;\sum_{i=1}^{N}{{\text{v}}^{*}}_{i}/N\right\|}^{2}.$$

The vector $\sum_{i=1}^{N}{{\text{v}}^{*}}_{i}/N$ is the mean of the sample. Thus, the nearest balance to the vector of the mean change is the least-squares estimate of the mean change among all balance vectors.

### (ii) Least-squares estimate for linear regression with a single predictor.

For the case of a single predictor, the linear regression and the single-balance model are written as

$${{\text{y}}^{*}}_{i}\;\sim \;N({{\text{a}}^{*}+\text{x}}_{i}{\text{v}}^{*}\text{,}\;\sigma^{2}I\text{)}$$

and

$${{\text{y}}^{*}}_{i}\;\sim \;N({{\text{a}}^{*}}_{\mathrm{sb}}+{\text{x}}_{i}{\text{b}}^{*}\text{,}\;\sigma_{\mathrm{sb}}^{2}I\text{)}.$$

*Theorem.* Let v*_l_**_s_** be the least-squares estimate of v*, β_ls_ be nearest to v*_ls_ balance vector and ${\text{a}}_{\text{ls}}={\bar{y}}^{*}-\bar{x}\beta_{\text{ls}}$, where ${\bar{y}}^{*}=\sum_{i=1}^{N}{\text{y}}_{i}/N$ and $\bar{x}=\sum_{i=1}^{N}{\text{x}}_{i}/N$ are the observation and predictor means. Then [β_ls_, a_ls_] is the least-squares estimate of the single-balance regression coefficients [b*, a*_sb_].

*Proof.* For an arbitrary estimate of single-balance regression coefficients the residual sum of squares (RSS) is as follows:

$$\text{RSS}\left(\text{a,}\beta \right)=\sum_{i=1}^{N}\|{{\text{y}}^{*}}_{i}\;-\text{ a }-{\text{ x}}_{i}{\beta \|}^{2}=\sum_{i=1}^{N}{\left({{\text{y}}^{*}}_{i}\;-\text{ a }-{\text{ x}}_{i}\beta \right)}^{T}\left({{\text{y}}^{*}}_{i}\;-\text{ a }-{\text{ x}}_{i}\beta \right)$$

Zero value of partial derivatives of the function by a* is a necessary condition of its minimum:

$$\frac{\partial \text{RSS}}{\partial \text{a}}\left({\text{a}}_{\text{ls}},\beta_{\mathrm{ls}}\right)=-2\sum_{i=1}^{N}\left({{\text{y}}^{*}}_{i}\;-{\text{ x}}_{i}\beta_{\mathrm{ls}}\right)+2N{\text{a}}_{\text{ls}}=0,$$

where 0 is a zero vector. This leads to a relation between the coordinates of the least-squares estimates of the coefficients:

$${\text{a}}_{\text{ls}}=\;{\bar{y}}^{*}\;-\;\bar{x}\beta_{\text{ls}}.$$

Substituting the expression to RSS leads to requirement

$$\beta_{\mathrm{ls}}=\underset{\beta \in B^{D-1}}{\text{argmin}}\sum_{i=1}^{N}{\left\|\delta {{\text{y}}^{*}}_{i}\;-\;\delta {\text{x}}_{i}\beta \right\|}^{2}=$$

$$=\underset{\beta \in B^{D-1}}{\text{argmin}}\sum_{i=1}^{N}\left[{\left(\delta {{\text{y}}^{*}}_{i}\right)}^{T}\delta {{\text{y}}^{*}}_{i}\;+\;{\left(\delta {\text{x}}_{i}\right)}^{2}\beta^{T}\beta \;-\;2\delta {\text{x}}_{i}\beta^{T}\delta {{\text{y}}^{*}}_{i}\right]=$$

$$=\underset{\beta \in B^{D-1}}{\text{argmin}}\|\beta \;-\;{\left(\sum_{i=1}^{N}\delta {\text{x}}_{i}\delta {{\text{y}}^{*}}_{i}\right)/\left(\sum_{i=1}^{N}{\left(\delta {\text{x}}_{i}\right)}^{2}\right)\|}^{2}$$

where $\delta {{\text{y}}^{*}}_{i}={{\text{y}}^{*}}_{i}-{\bar{y}}^{*}$ and $\delta {\text{x}}_{i}={\text{x}}_{i}–\bar{x}$ are deviations of the observation and predictor values from the means. Here, we do not use a partial derivative the way it was done for ∂*RSS*/∂a. The reason is that β is restricted to be a balance vector; thus, *RSS* is not a continuous function of β.

Similar calculations give the well-known least-squares estimate of the ordinary linear regression coefficient v*:

$${{\text{v}}^{*}}_{\mathrm{ls}}=\underset{v*\in {\mathbb{R}}^{D-1}}{\text{argmin}}{\left\|{\text{v}}^{*}\;-\;\left(\sum_{i=1}^{N}\delta {\text{x}}_{i}\delta {{\text{y}}^{*}}_{i}\right)/\left(\sum_{i=1}^{N}{\left(\delta {\text{x}}_{i}\right)}^{2}\right)\right\|}^{2}=$$

$$=\left(\sum_{i=1}^{N}\delta {\text{x}}_{i}\delta {{\text{y}}^{*}}_{i}\right)/\left(\sum_{i=1}^{N}{\left(\delta {\text{x}}_{i}\right)}^{2}\right)$$

Thus,

$${\text{a}}_{\text{ls}}={\bar{y}}^{*}\;-\;\bar{x}\beta_{\text{ls}},$$

$$\beta_{\text{ls}}=\underset{\beta \in B^{D\;-\;1}}{\text{argmin}}{\left\|\beta -{{\text{v}}^{*}}_{\text{ls}}\right\|}^{2}$$

The last equation means that β_ls_ is the nearest to the v*_ls_ vector.

*End of the proof*.

### Data sets. (i) Simulated data for exploitation of stability of the nearest balance method.

Microbiome composition profiles of stool samples collected before and after short-term high-fiber dietary intervention were taken as a start point (26) (*n* = 368). Three types of filtration were applied to the data: taxa present at the level of ≥2 reads in ≥5, 20, or 50% of samples were included in the analysis. Zeros replacement was done with the *cmultRepl*() function from the *zCompositions* package. Principal balance analysis using the hierarchical clustering of components method was applied to construct an ILR system of coordinates. The mean shift in the coordinates was used as the disturbed vector. Noise was simulated from a multivariate normal distribution with zero mean and identity covariate matrix in the subspace orthogonal to the mean shift. The noise was resized to a certain length: 5%, 10%, 20%, or 30% of the mean shift length. The disturbed vector was calculated as the sum of the mean shift and the noise. For each noise size, 20 disturbed vectors were generated.

### (ii) Simulated data for cross-validation.

A case-control study observations were simulated for cross-validation. The HIV data set (27) included in the *selbal* package was used as the ground for simulation. The low-abundance taxa were filtered from the read counts table; those with >15 reads in >30% of samples were included in the analysis. An ILR system of coordinates was constructed by the PDBA method from the *balance* package. A multivariate linear regression with HIV_Status and MSM predictors was fitted in these coordinates. The prediction of the model for subjects with negative HIV status and “nonMSM” value of MSM factor were used as the mean microbiome composition for one of the simulated groups. The mean value in the other group was obtained by a shift of a single balance in the direction of one of the balancing elements of the ILR system. The size of the shift was equal to the one associated with the MSM factor estimated by the regression model. One thousand samples were simulated for each of the groups from the multivariate normal distribution with dispersion parameter from the linear regression model.

### (iii) CD data sets.

The reads for stool samples of healthy subjects and subjects with Crohn’s disease were taken from references 28 and 12. In reference 28, only samples from the Spanish cohort collected at the first time point were included (34 healthy subjects versus 34 patients with CD). In reference 12, only the samples from the Chinese cohort with a single sample per subject were used, and patients under infliximab treatment were excluded from the analysis. The tables of counts were obtained as described previously (29). Only taxa that were present at a level of >5 reads in ≥50% of samples of the Spanish cohort were included in the analysis. The zero replacement was done with the *cmultRepl*() function from the *zCompositions* package.

### Data availability.

The code for simulations and cross-validation is available at https://bitbucket.org/knomics/nearest_balance_for_paper. The algorithms are implemented in the R library NearestBalance (https://bitbucket.org/knomics/nearestbalance).

10.1128/msystems.00155-22.7 FIG S4 Example of a tree obtained by algorithm A3. Tree for the CLR vector with elements [v̂_1_, v̂_2_, v̂_3_, v̂_4_, v̂_5_, v̂_6_, v̂_7_, v̂_8_] = [−0.1, −0.8, −0.1, −0.3, 0.1, 0.9, −0.6, 0.9] obtained with the nearest balance algorithm. The fill color denotes the impact of each balance. Download FIG S4, EPS file, 0.06 MB. Copyright © 2022 Odintsova et al. 2022 Odintsova et al. https://creativecommons.org/licenses/by/4.0/ This content is distributed under the terms of the Creative Commons Attribution 4.0 International license.

10.1128/msystems.00155-22.8 TABLE S1 Example of the balances iteratively obtained by algorithm A3. Shown are the balances for the CLR vector with components [v̂_1_, v̂_2_, v̂_3_, v̂_4_, v̂_5_, v̂_6_, v̂_7_, v̂_8_] = [−0.1, −0.8, −0.1, −0.3, 0.1, 0.9, −0.6, 0.9]. Download Table S1, DOCX file, 0.01 MB. Copyright © 2022 Odintsova et al. 2022 Odintsova et al. https://creativecommons.org/licenses/by/4.0/ This content is distributed under the terms of the Creative Commons Attribution 4.0 International license.

10.1128/msystems.00155-22.9 TABLE S2 Example of algorithm A3 implementation: stage 2. For brevity, only some combinations of *r* and *s* are shown. Download Table S2, DOCX file, 0.01 MB. Copyright © 2022 Odintsova et al. 2022 Odintsova et al. https://creativecommons.org/licenses/by/4.0/ This content is distributed under the terms of the Creative Commons Attribution 4.0 International license.

10.1128/msystems.00155-22.10 TABLE S3 Example of algorithm A3 implementation: stage 7. For brevity, only some combinations of *r* and *s* are shown. Download Table S3, DOCX file, 0.01 MB. Copyright © 2022 Odintsova et al. 2022 Odintsova et al. https://creativecommons.org/licenses/by/4.0/ This content is distributed under the terms of the Creative Commons Attribution 4.0 International license.
