# Supplementary material for: Approximation of a Microbiome Composition Shift by a Change in a Single Balance Between Two Groups of Taxa
Source: mSystems. 2022 May 9;7(3):e00155-22. doi: 10.1128/msystems.00155-22 (PMC9239069; doi:10.1128/msystems.00155-22)
Supplement: TEXT S1 [file msystems.00155-22-s0001.docx]

**SUPPLEMENTAL NOTE 1**

*Statement*. Let **w*** be an arbitrary vector and **b*_1_** - a balance vector in ILR coordinates. Then a balance vector **b*_2_** is the nearest balance to **w*** among all vectors orthogonal to **b*_1_** if and only if **b*_1_ + b*_2_** is the nearest to **w*** among all sums of **b_1_*** with a balance vector**.**

*Proof.* Let **w*** be an ILR-vector. As **b*_1_** and **b*_2_** are orthogonal, **b*_1_**^T^**b*_2_** = 0. Thus,

$\underset{\{\mathbf{b}_{\boldsymbol{2}}\in\mathcal{B}^{D-1} |\mathbf{b}_{\boldsymbol{2}}\perp\mathbf{b}_{\boldsymbol{1}}\}}{\mathrm{argmin}} \left\| {\mathbf{b}\text{*}}_{1}+{\mathbf{b}\text{*}}_{2}-\mathbf{w}\text{*} \right\|^{2}$ =

$$=\underset{\{\mathbf{b}_{\boldsymbol{2}}\in\mathcal{B}^{D-1} |\mathbf{b}_{\boldsymbol{2}}\perp\mathbf{b}_{\boldsymbol{1}}\}}{\mathrm{argmin}} \left( {\mathbf{b}\text{*}}_{1}+{\mathbf{b}\text{*}}_{2}-\mathbf{w}\text{*} \right)^{T}\left( {\mathbf{b}\text{*}}_{1}+{\mathbf{b}\text{*}}_{2}-\mathbf{w}\text{*} \right)=$$

$$=\underset{\{\mathbf{b}_{\boldsymbol{2}}\in\mathcal{B}^{D-1} |\mathbf{b}_{\boldsymbol{2}}\perp\mathbf{b}_{\boldsymbol{1}}\}}{\mathrm{argmin}} \left[ \left( {\mathbf{b}\text{*}}_{1}-\mathbf{w}\text{*} \right)^{T}\left( {\mathbf{b}\text{*}}_{1}-\mathbf{w}\text{*} \right)+2\left( {\mathbf{b}\text{*}}_{1}-\mathbf{w}\text{*} \right)^{T}{\mathbf{b}\text{*}}_{2} +{{\mathbf{b}\text{*}}_{\boldsymbol{2}}}^{\boldsymbol{T}}{\mathbf{b}\text{*}}_{\boldsymbol{2}} \right]=$$

$$=\underset{\{\mathbf{b}_{\boldsymbol{2}}\in\mathcal{B}^{D-1} |\mathbf{b}_{\boldsymbol{2}}\perp\mathbf{b}_{\boldsymbol{1}}\}}{\mathrm{argmin}} \left[ \left( {\mathbf{b}\text{*}}_{1}-\mathbf{w}\text{*} \right)^{T}\left( {\mathbf{b}\text{*}}_{1}-\mathbf{w}\text{*} \right)-2{\mathbf{w}\text{*}}^{T}{\mathbf{b}\text{*}}_{1} +{{\mathbf{b}\text{*}}_{\boldsymbol{2}}}^{\boldsymbol{T}}{\mathbf{b}\text{*}}_{\boldsymbol{2}} \right]=$$

$$=\underset{\{\mathbf{b}_{\boldsymbol{2}}\in\mathcal{B}^{D-1} |\mathbf{b}_{\boldsymbol{2}}\perp\mathbf{b}_{\boldsymbol{1}}\}}{\mathrm{argmin}} \left[ \left( {\mathbf{b}\text{*}}_{1}-\mathbf{w}\text{*} \right)^{T}\left( {\mathbf{b}\text{*}}_{1}-\mathbf{w}\text{*} \right)-2{\mathbf{w}\text{*}}^{T}{\mathbf{b}\text{*}}_{1} +{{\mathbf{b}\text{*}}_{\boldsymbol{2}}}^{\boldsymbol{T}}{\mathbf{b}\text{*}}_{\boldsymbol{2}} \right]=$$

$=\underset{\{\mathbf{b}_{\boldsymbol{2}}\in\mathcal{B}^{D-1} |\mathbf{b}_{\boldsymbol{2}}\perp\mathbf{b}_{\boldsymbol{1}}\}}{\mathrm{argmin}} \left[ \left\| {\mathbf{b}\text{*}}_{1}-\mathbf{w}\text{*} \right\|^{2} + \left\| {\mathbf{b}\text{*}}_{2}-\mathbf{w}\text{*} \right\|^{2}{+ \left\| \mathbf{w}\text{*} \right\|}^{2} \right]=$

$=\underset{\{\mathbf{b}_{\boldsymbol{2}}\in\mathcal{B}^{D-1} |\mathbf{b}_{\boldsymbol{2}}\perp\mathbf{b}_{\boldsymbol{1}}\}}{\mathrm{argmin}} \left\| {\mathbf{b}\text{*}}_{2}-\mathbf{w}\text{*} \right\|^{2}$

*End of the proof.*
