# Supplementary material for: Approximation of a Microbiome Composition Shift by a Change in a Single Balance Between Two Groups of Taxa
Source: mSystems. 2022 May 9;7(3):e00155-22. doi: 10.1128/msystems.00155-22 (PMC9239069; doi:10.1128/msystems.00155-22)
Supplement: TEXT S3 [file msystems.00155-22-s0003.docx]

**SUPPLEMENTAL NOTE 3**

Below is an example of constructing the tree for the CLR-vector **v̂** with elements [v̂_1_, v̂_2_, v̂_3_, v̂_4_, v̂_5_, v̂_6_, v̂_7_, v̂_8_] = [-0.1, -0.8, -0.1, -0.3, 0.1, 0.9, -0.6, 0.9] using the nearest balance algorithm. The resultant tree is shown on Fig. S4 and in Table S1. We will consider a step-by-step algorithm for obtaining a tree, filling in successively the rows of Table S1. The balances that we will receive at each step will consist of numerator (+) and denominator (‒) parts representing elements of the vector **v̂** or from the balances obtained at the previous steps.

1. Input variable is a vector **v̂** in the CLR space with components **v̂ =** [v̂_1_, v̂_2_, v̂_3_, v̂_4_, v̂_5_, v̂_6_, v̂_7_, v̂_8_] = [-0.1, -0.8, -0.1, -0.3, 0.1, 0.9, -0.6, 0.9] **(Fig. S3, step s1)**.
2. The nearest balance is calculated by algorithm A1 (Fig. S3, step s2): order the elements of **v̂** and calculate cos(α) only once for each *r* and *s* combinations (*r* = 1, …, 7; *s* = 1, …, 7). By including *r* top and *s* bottom values from the sorted list, we will obtain max cos(a) for the fixed *r* and *s* values. Sorted **v̂** components are [v̂_8_, v̂_6_, v̂_5_, v̂_3_, v̂_1_, v̂_4_, v̂_7_, v̂_2_] = [ 0.9, 0.9, 0.1, -0.1, -0.1, -0.3, -0.6, -0.8]. Max cos(α) for each *r* and *s* combinations are calculated as described in A1 (Table S2).
3. For **v̂^+^** there are only 2 elements, therefore there is no need to resolve inner structure. So, we are starting to fill the result Table S2 filling it’s first line (bal1) **(Fig. S3, steps s5, s6)**.
4. The **v̂**^‒^ inner structure can be resolved by calculating the nearest vector to [v̂_2_, v̂_7_, v̂_4_] with the algorithm A1 (r = 1, 2; s=1, 2). Max cos(a) is observed with r = 1, s = 2 and **v̂** ^‒ +^ = [v̂_7_]; **v̂** ^‒ ‒^ = [v̂_2_, v̂_4_]. With these values we populate the 2nd and 3d lines of the Table S2 with bal2 and bal3 **(Fig. S3, steps s5, s6)**.
5. All branches of the balance calculated in stage 2 of this example are resolved. Now we can add this balance to the Table S1 by filling its 4th line (bal4) **(Fig. S3, step s7)**.
6. We have finished working with the internal balance structure of the bal4. Now we can return to the remaining part of the tree. First of all, we need to replace bal4 elements with one value bal4_mean = (v̂_2_+v̂_4_+v̂_6_+v̂_7_+v̂_8_)/5 = 0.02 **(Fig. S3, steps s8, s9)**. New vector v̂՛ will have the following elements **v̂՛** = [v̂_1_, v̂_3_, v̂_5_, bal4_mean] = [-0.1, -0.1, 0.1, 0.02]. The cosine value may be calculated using weighted sums of **v̂**՛ elements:

$cos\left( \alpha\right) =\sqrt{\frac{rs}{r+s}\left\{ \frac{1}{r}\left[ {\hat{v}՛}_{1}^{+}w_{1}^{+}+...+{\hat{v}՛}_{R}^{+}w_{R}^{+} \right]-\frac{1}{s}\left[ {\hat{v}՛}_{1}^{-}w_{1}^{-}+...+{\hat{v}՛}_{S}^{-}w_{S}^{-} \right] \right\}}$,

where $w_{1}^{+}$+ … + $w_{R}^{+}$ = *r*, and $w_{1}^{-}$+ … + $w_{S}^{-}=s$. In our case, weights are w = [1, 1, 1, 5]. For example, the balance between v̂_1_ and bal4_mean will be calculated as follows:

$$\sqrt{\frac{1\cdot5}{1+5}}\left\{ \hat{v}_{1}-\frac{1}{5}\left[ bal4\_mean\cdot5 \right] \right\}=\sqrt{\frac{1\cdot5}{1+5}}\left\{ \hat{v}_{1}-\frac{1}{5}\left[ \hat{v}_{2}+{\hat{v}_{4}+\hat{v}}_{6}+\hat{v}_{7}+\hat{v}_{8} \right] \right\}.$$

1. As the elements of **v̂՛** have in general unequal weights, we cannot directly apply the algorithm A1 used in stage 2 of this example. Now for each *r* and s combinations (*r*=1, …,7; *s*=1, …, 7) we must find all combinations of weights by which they can be obtained and then calculate $cos\left( \alpha\right)$. Firstly, we will sort the **v̂՛** vector for each unique weight. Sorted **v̂՛** for weights equal to 1 will be [v̂_1_, v̂_3_, v̂_5_] = [-0.1, -0.1, 0.1]; for weights equal to 5 - [bal4_mean] = [0.02]. Local max cos(a) for each *r* and *s* combinations are shown in Table S3. Max $\cos\left( \alpha\right)$ is observed with *r* = 1, *s* = 2 and **v̂՛^+^** = [v̂5]; **v̂՛^‒^** = [v̂1, v̂3] **(Fig. S3, iteration 2, step s2)**.
2. For **v̂՛^+^** there is only 1 element, so there is no need to resolve inner structure or add new balances to the resulting Table S1 **(Fig. S3, iteration 2, steps s4, s5, s6)**.
3. For v̂՛^‒^ there are only 2 elements, no need to resolve inner structure. We add balance to the resulting Table S1 filling the line 5 (bal5) **(Fig. S3, iteration 2, steps s4, s5, s6)**.
4. All branches of the balance calculated in stage 7 are resolved. Now we can add the balance to the result table filling the line 6 in the resulting Table S1 (bal6) **(Fig. S3, iteration 2, step s7)**.
5. New vector **v̂՛՛** is calculated. Elements from bal6 are substituted by one coordinate: bal6_mean = (v̂1+v̂3+v̂5)/3 = -0.03. We obtain **v̂՛՛** = [bal4_mean, bal6_mean] = [0.02, -0.03] with the weights w՛՛ = [5, 3] **(Fig. S3, iteration 2, steps s8, s9)**.
6. For **v̂՛՛** there are only 2 elements, no need to resolve structure. We can add balance to the resulting Table S1 filling the line 7 (bal7). This is the end of the tree calculation.
