# Supplementary figures and images for: Approximation of a Microbiome Composition Shift by a Change in a Single Balance Between Two Groups of Taxa
Source: mSystems. 2022 May 9;7(3):e00155-22. doi: 10.1128/msystems.00155-22 (PMC9239069; doi:10.1128/msystems.00155-22)

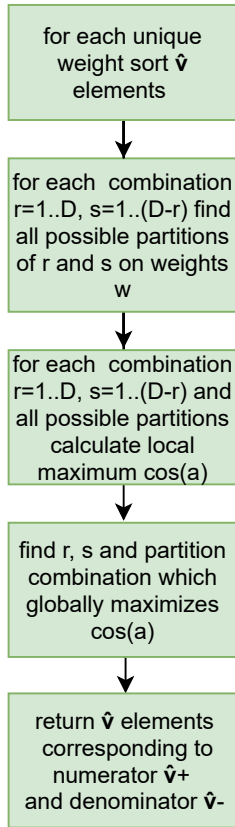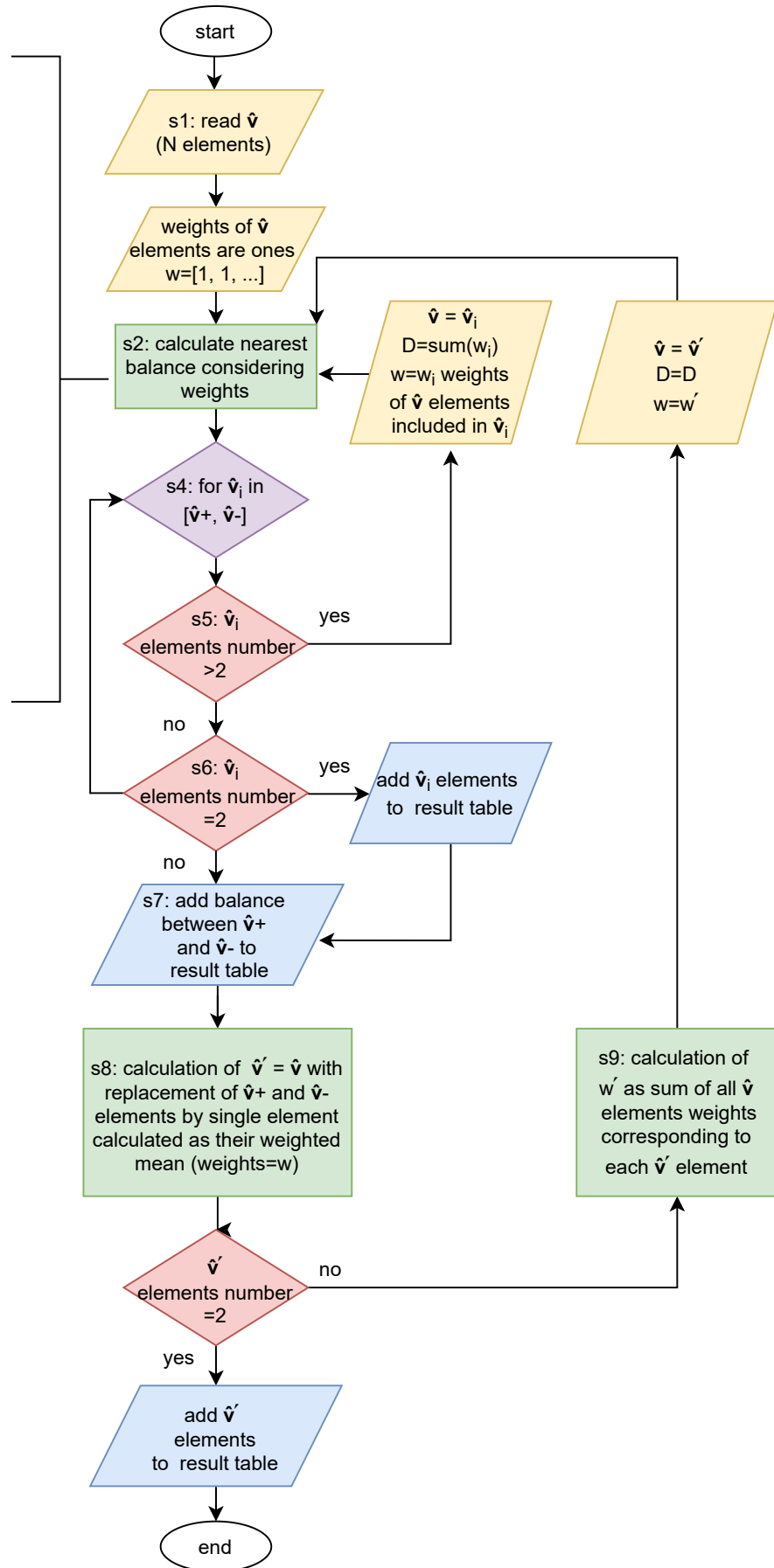

Supplement: FIG S3 [file msystems.00155-22-s0006.pdf]
