# Supplementary material for: Approximation of a Microbiome Composition Shift by a Change in a Single Balance Between Two Groups of Taxa
Source: mSystems. 2022 May 9;7(3):e00155-22. doi: 10.1128/msystems.00155-22 (PMC9239069; doi:10.1128/msystems.00155-22)
Supplement: TEXT S2 [file msystems.00155-22-s0002.docx]

**SUPPLEMENTAL NOTE 2**

Following notations in the main text, the multivariate linear regression model assumes that

**y***_i_ ~ N(x_i_**v***+ A*z_i_, 𝜎^2^𝕀),

and the single-balance linear regression assumes that

**y***_i_ ~ N(x_i_**b*** + A*_sb_ z_i_, 𝜎_sb_^2^𝕀).

*Theorem.* Let **v*_ls_** be the least squares estimate of coefficient **v***, **β_ls_** be the balance vector nearest to **v*_ls_**, 𝒜_ls_ = (Y* - **β_ls_**X^T^)Z^T^(ZZ^T^)^-1^. Then [**β_ls_** , 𝒜_ls_] is the least squares estimate of the single-balance regression coefficients [**b***, A*_sb_].

*Proof.* For an arbitrary estimate [**β**, 𝒜] of coefficients [**b***, A*_sb_], the residual sum of squares (RSS) of the single-balance model is

RSS(**β**, 𝒜) = $\sum_{i=1}^{N} {\|{\mathbf{y}\text{*}}_{i}-\boldsymbol{\beta}x_{i}-A\text{*}z_{i}\|}^{2}$ = $\sum_{i=1}^{N} {({\mathbf{y}\text{*}}_{i}-\boldsymbol{\beta}x_{i}-A\text{*}z_{i})}^{T}({\mathbf{y}\text{*}}_{i}-\boldsymbol{\beta}x_{i}-A\text{*}z_{i})$

The least squares solution [**β**, 𝒜] of the single-balance regression model coefficients is the argmin of this expression. For fixed **β**, the minimizer 𝒜 of RSS must provide zero value of ${\partial\mathrm{RSS}}/{\partial\mathcal{A}}$. Thus, least squares estimate of coefficients should obey the equation

2(𝒜_ls_Z - Y* + **β_ls_**X)Z*^T^* = 0.

and the least squares estimate of A*_sb_ obeys

𝒜_ls_(**β_ls_**) = (Y* - **β_ls_**X)Z^T^(ZZ^T^)^-1^**_._**

Thus,

$\boldsymbol{\beta}_{\mathbf{ls}}=\underset{\boldsymbol{\beta}\in\mathcal{B}^{D-1}}{\mathrm{argmin}} \sum_{i=1}^{N} {({\mathbf{y}\text{*}}_{i}-\boldsymbol{\beta}x_{i}-{\mathcal{A}_{\mathrm{ls}}(\boldsymbol{\beta})z}_{i})}^{T}({\mathbf{y}\text{*}}_{i}-\boldsymbol{\beta}x_{i}-\mathcal{A}_{\mathrm{ls}}(\boldsymbol{\beta})z_{i})=$

$=\underset{\boldsymbol{\beta}\in\mathcal{B}^{D-1}}{\mathrm{argmin}} \sum_{i=1}^{N} {(\delta{\mathbf{y}\text{*}}_{i}-\boldsymbol{\beta}\delta x_{i})}^{T}(\delta{\mathbf{y}\text{*}}_{i}-\boldsymbol{\beta}\delta x_{i})=$

$=\underset{\boldsymbol{\beta}\in\mathcal{B}^{D-1}}{\mathrm{argmin}} \sum_{i=1}^{N} \left[ {(\delta{\mathbf{y}\text{*}}_{i})}^{T}(\delta{\mathbf{y}\text{*}}_{i})-2{(\delta{\mathbf{y}\text{*}}_{i})}^{T}\boldsymbol{\beta}\delta x_{i}+{(\boldsymbol{\beta}\delta x_{i})}^{T}\boldsymbol{\beta}\delta x_{i} \right]$

where 𝛿x*_i_* = x*_i_* - X^T^Z^T^(ZZ^T^)^-1^z*_i_*, $\delta{\mathbf{y}\text{*}}_{i}$**= y****_i_* - YZ^T^(ZZ^T^)^-1^z*_i_*. As the first term does not depend on **β** and 𝛿x*_i_* is scalar,

$\boldsymbol{\beta}_{\mathbf{ls}}=\underset{\boldsymbol{\beta}\in\mathcal{B}^{D-1}}{\mathrm{argmin}} \sum_{i=1}^{N} \left[ {(\delta x_{i})}^{2}\boldsymbol{\beta}^{T}\boldsymbol{\beta}-2{\delta x_{i}(\delta{\mathbf{y}\text{*}}_{i})}^{T}\boldsymbol{\beta} \right] =$

$=\underset{\boldsymbol{\beta}\in\mathcal{B}^{D-1}}{\mathrm{argmin}} \left[ \boldsymbol{\beta}^{T}\boldsymbol{\beta} -2\left( {\sum_{i=1}^{N} {\delta x_{i}(\delta{\mathbf{y}\text{*}}_{i})}^{T}}/{\sum_{i=1}^{N} {(\delta x_{i})}^{2}} \right)\boldsymbol{\beta} \right] =$

$=\underset{\boldsymbol{\beta}\in\mathcal{B}^{D-1}}{\mathrm{argmin}} \left[ \boldsymbol{\beta} -\left( {\sum_{i=1}^{N} {\delta x_{i}(\delta{\mathbf{y}\text{*}}_{i})}^{T}}/{\sum_{i=1}^{N} {(\delta x_{i})}^{2}} \right) \right]^{T}\left[ \boldsymbol{\beta} -\left( {\sum_{i=1}^{N} {\delta x_{i}(\delta{\mathbf{y}\text{*}}_{i})}^{T}}/{\sum_{i=1}^{N} {(\delta x_{i})}^{2}} \right) \right]$

Above we use the fact that adding a constant to a function or multiplying it by a constant does not change its argmin. Hence,

$\boldsymbol{\beta}_{\mathbf{ls}}=\underset{\boldsymbol{\beta}\in\mathcal{B}^{D-1}}{\mathrm{argmin}} \left\| \boldsymbol{\beta} -\left( {\sum_{i=1}^{N} {\delta x_{i}(\delta{\mathbf{y}\text{*}}_{i})}^{T}}/{\sum_{i=1}^{N} {(\delta x_{i})}^{2}} \right) \right\|^{2}$

Similar calculations show that the least squares estimate of the ordinary linear regression model is

${\mathbf{v}\text{*}}_{\mathbf{ls}}=\underset{v*\in\mathbb{R}^{D-1}}{\mathrm{argmin}} \left\| \mathbf{v}\text{*} -\left( {\sum_{i=1}^{N} {\delta x_{i}(\delta{\mathbf{y}\text{*}}_{i})}^{T}}/{\sum_{i=1}^{N} {(\delta x_{i})}^{2}} \right) \right\|^{2}$ =

$={\sum_{i=1}^{N} {\delta x_{i}(\delta{\mathbf{y}\text{*}}_{i})}^{T}}/{\sum_{i=1}^{N} {(\delta x_{i})}^{2}}$

Thus,

$$\boldsymbol{\beta}_{\mathbf{ls}}=\underset{\boldsymbol{\beta}\in\mathcal{B}^{D-1}}{\mathrm{argmin}} \left\| \boldsymbol{\beta} -{\mathbf{v}\text{*}}_{\mathbf{ls}} \right\|^{2}$$

i.e., **β_ls_** is the nearest to **v*_ls_** balance and

𝒜_ls_ = (Y* - **β_ls_**X^T^)Z^T^(ZZ^T^)^-1^

*End of the proof.*
