# Supplementary material for: Approximation of a Microbiome Composition Shift by a Change in a Single Balance Between Two Groups of Taxa
Source: mSystems. 2022 May 9;7(3):e00155-22. doi: 10.1128/msystems.00155-22 (PMC9239069; doi:10.1128/msystems.00155-22)
Supplement: TABLE S1 [file msystems.00155-22-s0007.docx]

**Table S1. An example of the balances iteratively obtained by algorithm A3.** Shown are the balances for the CLR vector with components [v̂_1_, v̂_2_, v̂_3_, v̂_4_, v̂_5_, v̂_6_, v̂_7_, v̂_8_] = [-0.1, -0.8, -0.1, -0.3, 0.1, 0.9, -0.6, 0.9].

| **Line** | **Balance ID** | **Numerator (+)** | **Denominator (-)** |
| --- | --- | --- | --- |
| 1 | bal1 | v̂_6_ | v̂_8_ |
| 2 | bal2 | v̂_4_ | v̂_2_ |
| 3 | bal3 | v̂_7_ | bal2 |
| 4 | bal4 | bal1 | bal3 |
| 5 | bal5 | v̂_1_ | v̂_3_ |
| 6 | bal6 | v̂_5_ | bal5 |
| 7 | bal7 | bal4 | bal6 |
