# Supplementary material for: Approximation of a Microbiome Composition Shift by a Change in a Single Balance Between Two Groups of Taxa
Source: mSystems. 2022 May 9;7(3):e00155-22. doi: 10.1128/msystems.00155-22 (PMC9239069; doi:10.1128/msystems.00155-22)
Supplement: TABLE S2 [file msystems.00155-22-s0008.docx]

**Table S2. Example of algorithm A3 implementation: stage 2.** For brevity, only some combinations of *r* and *s* are shown.

| *s* | *r* | **v̂^+^** = [v̂^+^*_1_,* …, v̂^+^*_r_*] | **v̂**^‒^ = [v̂^‒^*_1_,* …, v̂^‒^*_s_*] | $cos(\alpha)\vert\vert\hat{\mathbf{v}}\vert\vert$ |
| --- | --- | --- | --- | --- |
| 1 | 1 | v̂_2_ | v̂_8_ | 1.2 |
| 1 | 2 | v̂_2_ | v̂_8_, v̂_6_ | 1.4 |
| 1 | 3 | v̂_2_ | v̂_8_, v̂_6_, v̂_5_ | 1.2 |
| ... | | | | |
| 3 | 2 | v̂_2_, v̂_7_, v̂_4_ | v̂_8_, v̂_6_ | 1.6 |
| ... | | | | |
| 7 | 1 | v̂_2_, v̂_7_, v̂_4_, v̂_1_, v̂_3_, v̂_5_, v̂_6_ | v̂_8_ | 1.0 |
