# Supplementary material for: Approximation of a Microbiome Composition Shift by a Change in a Single Balance Between Two Groups of Taxa
Source: mSystems. 2022 May 9;7(3):e00155-22. doi: 10.1128/msystems.00155-22 (PMC9239069; doi:10.1128/msystems.00155-22)
Supplement: TABLE S3 [file msystems.00155-22-s0009.docx]

**Table S3. An example of algorithm A3 implementation: stage 7.** For brevity, only some combinations of *r* and *s* are shown.

| ***s*** | ***r*** | **possible weight variants** | **v̂՛^-^** | **v̂՛^+^** | $\cos\left( \boldsymbol{\alpha} \right)\left\vert\left\vert\hat{\mathbf{v}} \right\vert\right\vert$ |
| --- | --- | --- | --- | --- | --- |
| 1 | 1 | [1, 1] | v̂_1_ | v̂_5_ | 0.14 |
| ... | | | | | |
| 2 | 1 | [1+1, 1] | v̂_1,_ v̂_3_ | v̂_5_ | 0.16 |
| 2 | 2 | no possible weights | - | - | - |
| ... | | | | | |
| 7 | 1 | [5+1+1,1] | bal4_mean, v̂_1,_ v̂_3_ | v̂_5_ | 0.11 |
